# Epistasis regulates genetic control of cardiac hypertrophy

**DOI:** 10.21203/rs.3.rs-3509208/v1

**Published:** 2023-11-20

**Authors:** Qianru Wang, Tiffany M. Tang, Nathan Youlton, Chad S. Weldy, Ana M. Kenney, Omer Ronen, J. Weston Hughes, Elizabeth T. Chin, Shirley C. Sutton, Abhineet Agarwal, Xiao Li, Merle Behr, Karl Kumbier, Christine S. Moravec, W. H. Wilson Tang, Kenneth B. Margulies, Thomas P. Cappola, Atul J. Butte, Rima Arnaout, James B. Brown, James R. Priest, Victoria N. Parikh, Bin Yu, Euan A. Ashley

**Affiliations:** 1. Division of Cardiovascular Medicine, Department of Medicine, Stanford University, Stanford, CA, USA; 2. Department of Statistics, University of California, Berkeley, Berkeley, CA, USA; 3. Faculty of Informatics and Data Science, University of Regensburg, Regensburg, Germany; 4. Department of Pharmaceutical Chemistry, University of California, San Francisco, San Francisco, CA, USA; 5. Department of Cardiovascular and Metabolic Sciences, Lerner Research Institute, Cleveland Clinic, Cleveland, OH, USA; 6. Department of Cardiovascular Medicine, Heart Vascular and Thoracic Institute, Cleveland Clinic, Cleveland, OH, USA; 7. Division of Cardiovascular Medicine, Perelman School of Medicine, University of Pennsylvania, Philadelphia, PA, USA; 8. Hospital of The University of Pennsylvania, Philadelphia, PA, USA; 9. Bakar Computational Health Sciences Institute, University of California, San Francisco, San Francisco, CA, USA; 10. Chan Zuckerberg Biohub – San Francisco, San Francisco, CA, USA; 11. Division of Environmental Genomics and Systems Biology, Lawrence Berkeley National Laboratory, Berkeley, CA, USA; 12. Tenaya Therapeutics, San Francisco, CA, USA; 13. Department of Electrical Engineering and Computer Science, University of California, Berkeley, Berkeley, CA, USA; 14. Center for Computational Biology, University of California, Berkeley, Berkeley, CA, USA

## Abstract

The combinatorial effect of genetic variants is often assumed to be additive. Although genetic variation can clearly interact non-additively, methods to uncover epistatic relationships remain in their infancy. We develop low-signal signed iterative random forests to elucidate the complex genetic architecture of cardiac hypertrophy. We derive deep learning-based estimates of left ventricular mass from the cardiac MRI scans of 29,661 individuals enrolled in the UK Biobank. We report epistatic genetic variation including variants close to *CCDC141*, *IGF1R*, *TTN*, and *TNKS.* Several loci not prioritized by univariate genome-wide association analysis are identified. Functional genomic and integrative enrichment analyses reveal a complex gene regulatory network in which genes mapped from these loci share biological processes and myogenic regulatory factors. Through a network analysis of transcriptomic data from 313 explanted human hearts, we show that these interactions are preserved at the level of the cardiac transcriptome. We assess causality of epistatic effects via RNA silencing of gene-gene interactions in human induced pluripotent stem cell-derived cardiomyocytes. Finally, single-cell morphology analysis using a novel high-throughput microfluidic system shows that cardiomyocyte hypertrophy is non-additively modifiable by specific pairwise interactions between *CCDC141* and both *TTN* and *IGF1R*. Our results expand the scope of genetic regulation of cardiac structure to epistasis.

Heart disease is closely tied to the structure of the heart^1^. Heart failure, a syndrome characterized by increased pressure within, or decreased output from, the heart is influenced by structural features including atrial and ventricular chamber size and wall thickness^2–5^. Left ventricular hypertrophy – increased thickness of the left ventricle (LV) – can be the result of mendelian genetic diseases like hypertrophic cardiomyopathy^6^ but is also a complex phenotypic trait influenced by multiple factors, genetic and environmental. Progressive LV hypertrophy carries significant independent risk for incident heart failure, atrial arrhythmia, and sudden death^7–10^, highlighting the need to understand genetic determinants of cardiac phenotype.

Recent discoveries leveraging cardiac magnetic resonance imaging in the UK Biobank (UKBB) have revealed that cardiac structure is in part determined by complex genetics^11–14^. Common genetic variants, many located near genetic loci associated with dilated cardiomyopathy and heart failure, have been found to influence LV size and systolic function^11^. Further, specific genetic variants that influence LV trabeculation have been shown to impact systolic function and overall risk of cardiomyopathy^13^. However, these variants remain inadequate to explain the total heritable disease risk^15^. Indeed, common genetic variants rarely act independently and additively as modeled by most genome-wide association studies (GWAS)^16^. There is growing evidence to support a disease risk model in which multiple genes interact non-additively with each other through epistasis^17,18^. Recent work has shown that common genetic variation influences susceptibility and expressivity of hypertrophic cardiomyopathy^14^. This raises the possibility that common epistatic interactions drive cardiac phenotype, holding significant potential for uncovering disease mechanisms and developing potential therapeutic strategies.

Several computational and experimental challenges need to be resolved to allow robust identification of epistasis. First, the combinatorial nature of interactions makes an exhaustive search computationally intractable. To reduce the computational burden and ensure stable discoveries, we developed an approach based on signed iterative random forests^19,20^ to uncover higher-order (not limited to pairwise) nonlinear interactions in a computationally-tractable manner. Second, many previously reported epistatic relationships were not replicated^21,22^. To achieve more trustworthy results, we adhered to a new framework for veridical data science^23^, centered around the principles of predictability, computability, and stability (PCS) and the need for transparent documentation of decisions made in data analysis pipelines. A third challenge is the generally small effect size of common genetic variants^15,24^ which impedes both the data-driven discovery and functional validation of epistatic interactions. In human biobanks, recent advances in deep-learning-enabled phenotyping^25^ using cardiac magnetic resonance images have led to more refined phenotypes at larger scales. At the cellular level, high-throughput microfluidic technologies^26–28^ have been integrated with artificial intelligence-based image analysis of single cell morphology^29^ and human induced pluripotent stem cell-derived cardiomyocytes^30^, opening up new possibilities for rapid, label-free detection of the phenotypic consequences of genetic perturbation.

## Results

**Fig. 1:**
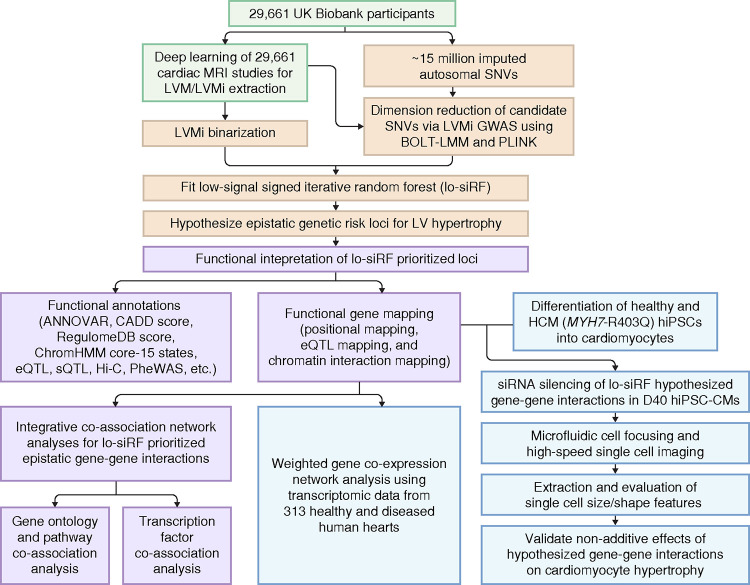
Schematic of the study workflow. The study workflow includes four major stages: (a) derivation of left ventricular mass from cardiac magnetic resonance imaging (green boxes); (b) computational prioritization of epistatic drivers (orange); (c) functional interpretation of the hypothesized epistatic genetic loci (purple); and (d) experimental confirmation of epistasis in cardiac tissues and cells (blue). Abbreviations: MRI, magnetic resonance imaging; LV, left ventricle; LVM, left ventricular mass; LVMi, left ventricular mass indexed by body surface area; SNV, single-nucleotide variant; GWAS, genome-wide association study; BOLT-LMM^35^ and PLINK^34^, two different GWAS software packages; lo-siRF, low-signal signed iterative random forest; ANNOVAR^78^, a software for functional annotation of genetic variants; CADD^40^, combined annotation dependent depletion, which scores the deleteriousness of variants; RegulomeDB^39^, a database that scores functional regulatory variants; ChromHMM^38^, a multivariate Hidden Markov Model for chromatin state annotation; eQTL, expression quantitative trait locus; sQTL, splicing quantitative trait locus; Hi-C, high-throughput chromosome conformation capture; PheWAS, phenome-wide association study; siRNA, small interfering RNA; hiPSC-CM, human induced pluripotent stem cell-derived cardiomyocyte; HCM, hypertrophic cardiomyopathy.

Our methodology includes four major stages: derivation of estimates of LV mass (green boxes, Fig. 1); computational prioritization of epistatic drivers (orange boxes, Fig.1); functional interpretation of the hypothesized epistatic genetic loci (purple boxes, Fig.1); and experimental confirmation of epistasis through perturbation (blue boxes, Fig. 1).

### Deep learning of UK Biobank cardiac imaging quantifies left ventricular hypertrophy

**Extended Data Fig. 1:**
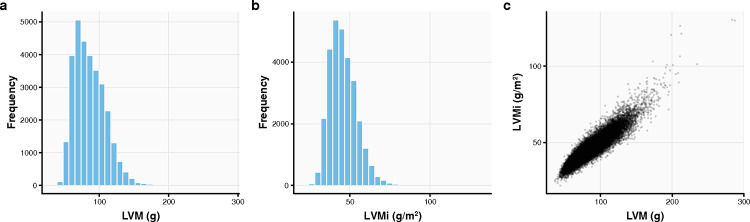
Distribution of LVM and LVMi measurements for 29,661 UK Biobank participants. Left ventricular mass (LVM, **a**) and LVM indexed to body surface area (LVMi, **b**) measurements were extracted from cardiac magnetic resonance imaging for 29,661 unrelated White British individuals via deep learning^25^. **c,** A high Pearson correlation of 0.92 was observed between these LVM and LVMi measurements.

**Fig. 2:**
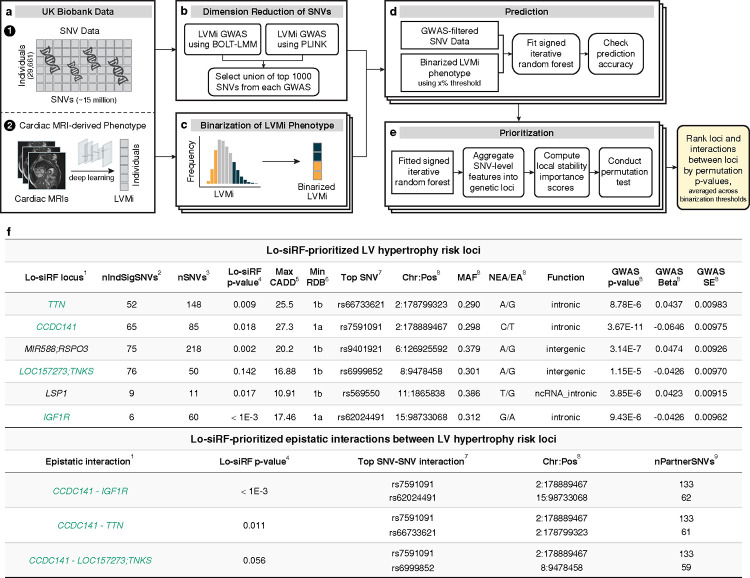
Low-signal signed iterative random forest (lo-siRF) prioritizes risk loci and epistatic interactions for left ventricular hypertrophy. **a-e**, Workflow of low-signal signed iterative random forest (lo-siRF). **a**, Lo-siRF took in as input single-nucleotide variant (SNV) data and cardiac MRI-derived left ventricular mass indexed by body surface area (LVMi) from 29,661 UK Biobank participants. **b**, Dimension reduction was performed via a genome-wide association study (GWAS) to concentrate the analysis on a smaller set of SNVs. **c**, LVMi was binarized into high and low LVMi categories according to three different binarization thresholds (represented by the stacked boxes). **d**, For each of the three binarization thresholds, a signed iterative random forest was fitted using the GWAS-filtered SNVs to predict the binarized LVMi phenotype. The validation prediction accuracy was assessed prior to interpreting the model fit. **e**, SNVs used in the fitted signed iterative random forest were aggregated into genetic loci based on annotations using ANNOVAR^78^. Genetic loci and pairwise interactions between loci were finally ranked according to their importance across the three signed iterative random forest fits, as measured by our proposed stability-driven importance score. **f**, Lo-siRF-prioritized risk loci and epistatic interactions. (1) Loci stably prioritized by lo-siRF as epistasis participants are highlighted in green. (2) nIndSigSNVs, the number of independent significant SNVs that are stably prioritized by lo-siRF across the three different LVMi binarization thresholds (panel **c**). (3) nSNVs, the number of candidate SNVs extracted by FUMA^37^ (v1.5.4) in strong LD (*r*^2^ > 0.6) with any of the lo-siRF-prioritized independent significant SNVs. (4) Lo-siRF *p*-value, the mean *p* value from lo-siRF, averaged across the three LVMi binarization thresholds. (5) Max CADD, the maximum CADD^40^ score of SNVs within or in LD with the specific locus. A high CADD score indicates a strong deleterious effect of the variant. A threshold of 12.37 has been suggested by Kircher et al.^40^. (6) Min RDB, the minimum RegulomeDB^39^ score of SNVs within or in LD with the specific locus. RDB is a categorical score to guide interpretation of regulatory variants (from 1a to 7, with 1a being the most biological evidence for an SNV to be a regulatory element)^37,39^. (7) The top-ranked SNV or SNV-SNV pair showing the highest occurrence frequency (Extended Data Fig. 4) averaged across lo-siRF fits from the three LVMi binarization thresholds. A full list of lo-siRF-prioritized SNVs and SNV-SNV pairs can be found in Extended Data 3. (8) Genomic location (hg38) and GWAS statistics information (using PLINK^34^) of the top SNV for each lo-siRF-prioritized locus. Abbreviations: MAF, minor allele frequency; NEA/EA, non-effect-allele/effect-allele; SE, standard error. (9) nPartnerSNVs, number of partner SNVs that interact with the given SNV in lo-siRF. These SNV-SNV pairs interacted in at least one lo-siRF decision path across every LVMi binarization threshold (details in Methods).

We accessed all cardiac magnetic resonance images from the UKBB substudy (44,503 people at the time of this analysis)^31^. We focused on the largest ancestry subset of 29,661 unrelated individuals (summary characteristics in Supplementary Table 1) and analyzed the most recent image per individual. We leveraged a recent deep learning model^25^ to quantify LV hypertrophy from these 29,661 multislice cine magnetic resonance images (Fig. 2a). A fully convolutional network had been previously trained for image segmentation and was evaluated on manual pixelwise-annotations of images from 4,875 UKBB participants^25^. This fully convolutional network learns features across five different resolutions through sequential convolutional layers interspersed with non-linearities, and has displayed accurate performance compared to cardiac segmentation by human experts^25^. Using this segmentation model, we extracted areas of the LV chamber wall in each slice of the short axis image at the end of diastole. Areas extracted from each image slice in the same image stack were then integrated to calculate the heart muscle volume, which we converted to the LV mass using a standard density of 1.05 g/mL^32^. This was normalized by body surface area, estimated using the Du Bois formula^33^, to obtain the LV mass index (LVMi, Extended Data Fig. 1). Details regarding this analysis can be found in Methods.

### Low-signal signed iterative random forests prioritizes epistatic genetic loci

**Extended Data Fig. 2:**
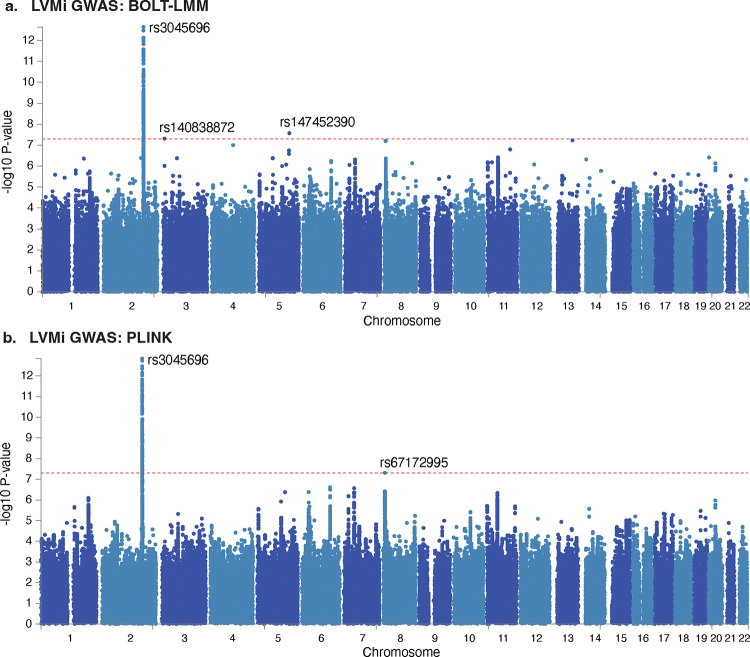
LVMi GWAS using BOLT-LMM and PLINK. GWAS using BOLT-LMM (**a**) and PLINK (**b**) identified associations with LVMi, of which the lead SNV rs3045696 showed the highest significance. This SNV rs3045696 was also identified as the top lead SNV by both BOLT-LMM and PLINK while other lead SNVs (labeled) were significant in either the BOLT-LMM GWAS or the PLINK GWAS but not both. The red dashed line denotes the genome-wide significance threshold (*p* < 5E-8). The two SNVs, rs3045696 and rs67172995, are also stably prioritized by lo-siRF as epistasis interactor variants. Details can be found in Extended Data 1.

**Extended Data Fig. 3:**
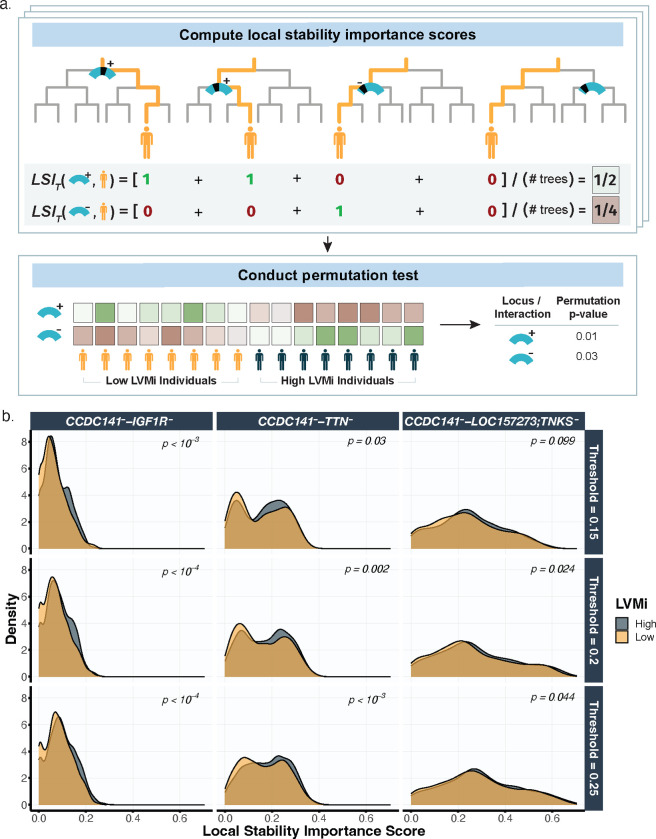
Differences in local stability scores between high and low LV mass highlight the importance of the lo-siRF-prioritized interactions between genetic loci. **a**, Schematic of local stability importance score computation. Given a locus (light blue transcript), the local stability importance score for an individual is defined as the proportion of trees for which at least one SNV (shaded black region) in the locus is used in the individual’s decision path. This computation (top) was performed for each individual (denoted by the stacked boxes). Then, a permutation test was conducted to assess the difference in these local stability importance scores between the low and high LVMi individuals (bottom). **b**, Differences in the distribution of local stability importance scores suggest that the identified interactions between genetic loci are important for differentiating individuals with high (dark gray) and low (orange) LVMi in the siRF fit. This result, evaluated on the validation data, is stable across the three binarization thresholds and is quantified by a permutation *p*-value given in the top right corner of each subplot.

We developed low-signal signed iterative random forests (lo-siRF, Fig. 2a–2e) to identify stable epistatic interactions from the extracted LV mass and single-nucleotide variants (SNVs) from UKBB. This method is guided by the PCS framework^23^ and builds upon signed iterative random forests^19,20^, a computationally-tractable algorithm to extract predictive and stable nonlinear higher-order interactions that frequently co-occur along decision paths in a random forest. More specifically, lo-siRF proceeds through four steps:

1. *Dimension reduction* (Fig. 2b): we combined the results of two initial genome-wide association studies, implemented via PLINK^34^ and BOLT-LMM^35^ (Extended Data Fig. 2, Extended Data 1) to reduce the interaction search space from 15 million imputed variants down to 1405 variants (Extended Data 2). Details can be found in the Methods section Lo-siRF step 1: Dimension reduction of variants via genome-wide association studies.
2. *Binarization* (Fig. 2c): we binarized the LV mass measurements into two (high and low) balanced categories (Supplementary Table 2). This binarization simplified the original low-signal regression problem for a (continuous) complex trait to a relatively easier classification task, which denoises and facilitates model checking. The detailed binarization procedure can be found in the Methods section Lo-siRF step 2: Binarization of the left ventricular mass phenotype.
3. *Prediction* (Fig. 2d): we trained a signed iterative random forest using the 1405 GWAS-filtered SNVs to predict the binarized LV mass measurements. The learnt model yields on average the highest (balanced) classification accuracy (55%) compared to other common machine learning prediction algorithms (Supplementary Table 3). Details about the model and prediction check can be found in the Methods section Lo-siRF step 3: Prediction.
4. *Prioritization* (Fig. 2e): we developed a stability-driven feature importance score (Extended Data Fig. 3), which leveraged the fitted signed iterative random forest and a permutation test, to aggregate SNVs into genetic loci and prioritize interactions between genetic loci. This importance score provides the necessary new interpretable machine learning ingredient to complete the lo-siRF discovery pipeline. Details can be found in the Methods section Lo-siRF step 4: Prioritization.

Extensive documentation and justification of our modeling decisions can be found in Supplementary Note 1, an interactive HTML webpage hosted at https://yu-group.github.io/epistasis-cardiac-hypertrophy/.

**Extended Data Fig. 4:**
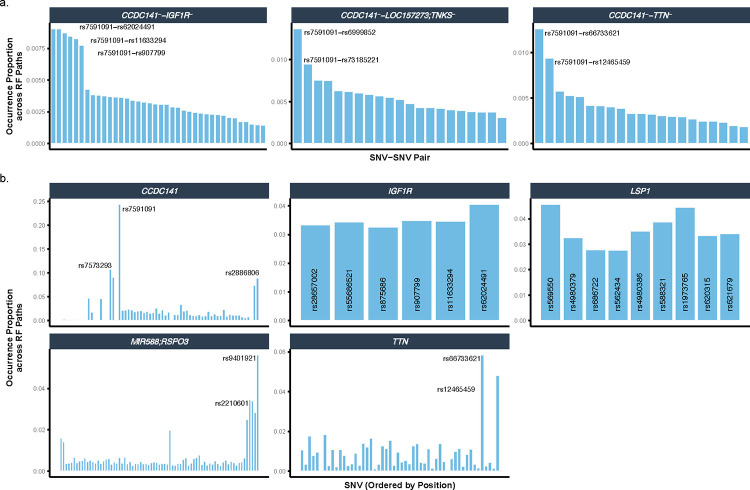
Top SNVs from lo-siRF-prioritized loci and interactions between loci. The most important SNVs and SNV-SNV pairs, as measured by their proportion of occurrence in the siRF fit are annotated for the top lo-siRF-prioritized interactions between loci in **a** and top genetic loci in **b**. The y-axis shows the proportion of decision paths in siRF, for which the SNV or SNV-SNV pair occurs, averaged across all three binarization thresholds. In each of the interactions between genetic loci, the SNV rs7591091 in the *CCDC141* locus appears most frequently, suggesting a key role in cardiac hypertrophy.

**Fig. 3:**
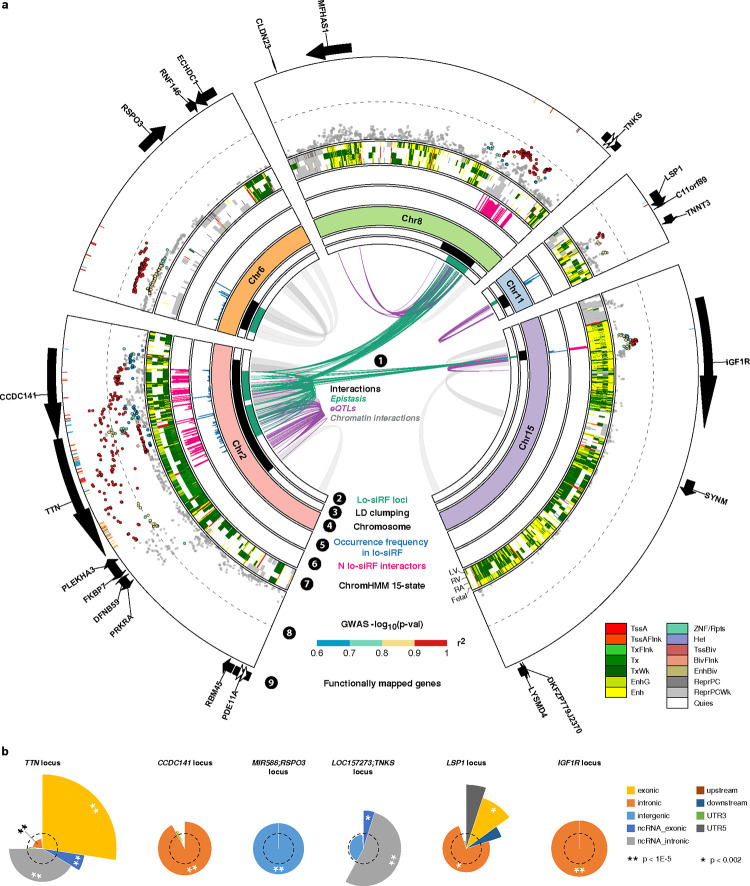
lo-siRF finds epistatic interactions between genetic risk loci for left ventricular hypertrophy. **a**, Circos plot showing the genetic risk loci identified by lo-siRF (green, circle 2) and regions after clumping FUMA-extracted SNVs in LD (*r*^2^ > 0.6) with lo-siRF-prioritized SNVs (black, circle 3). Circle 1 shows the top 300 epistatic SNV-SNV pairs with the highest frequency of occurrence in lo-siRF (green), SNV-gene linkages (FDR < 0.5) based on GTEx^41^ V8 cis-eQTL information from heart and skeletal muscle tissues (purple), and 3D chromatin interactions^37^ based on Hi-C data of left ventricular tissue obtained from GSE87112. Circle 5 and 6 show bar plots of the occurrence frequency and number of partner SNVs in epistasis (normalized by the maximum value of the corresponding locus) identified by lo-siRF, respectively. Circle 7 shows the ChromHMM^38^ core-15 chromatin state for left ventricle (LV), right ventricle (RV), right atrium (RA), and fetal heart (Fetal). Circle 8 shows the GWAS Manhattan plot from PLINK^34^ (circles) where only SNVs with *p* < 0.05 are displayed. The 283 lo-siRF-prioritized SNPs and their LD-linked (*r*^2^ > 0.6) SNVs are color-coded as a function of their maximum *r*^2^ value. A portion of these LD-linked SNPs (the outer heatmap layer in circle 8) are extracted from the selected FUMA reference panel (thereby with no GWAS *p*-values). SNVs that are not in LD (*r*^2^ ≤ 0.6) with any of the 283 lo-siRF-prioritized SNVs are gray. Dashed line indicates GWAS *p* = 5E-8. Circle 9 shows the 21 protein-coding genes mapped by FUMA. **b**, Pie charts showing ANNOVAR enrichment performed for each of the 6 lo-siRF loci (circle 2 in Fig. 3a and Fig. 2f). The arc length of each slice indicates the proportion of SNVs with a specific functional annotation. The radius of each slice indicates log_2_(E + 1), where E is the enrichment score computed as (proportion of SNVs with an annotation for a given locus)/(proportion of SNVs with an annotation relative to all available SNVs in the FUMA reference panel). The dashed circle indicates E = 1 (no enrichment). Asterisks indicate two-sided *p*-values of Fisher’s exact tests for the enrichment of each annotation. Details can be found in Extended Data 4 and 5.

Lo-siRF identified six genetic risk loci that exhibited stable and reliable associations with LV mass (Fig. 2f). Because these loci are either located within a gene body or in between two genes (Fig. 3a), for convenience we denote these loci by their nearest genes. Notably, out of the six loci, three (*TTN*, *CCDC141*, and *IGF1R*) were prioritized by lo-siRF as epistatic loci. These loci not only interact with other loci, but also marginally affect LV mass. The other three lo-siRF-prioritized loci are *LOC157273;TNKS*, *MIR588;RSPO3*, and *LSP1*. The *LOC157273;TNKS* locus is located within the intergenic region between genes *LOC157273* and *TNKS* (semicolon indicates intergenic region). This locus was prioritized by lo-siRF to be hypostatic (i.e., effects are deemed stable by lo-siRF only when interacting with the *CCDC141* locus). Interestingly, all three identified epistatic interactions involved the *CCDC141* locus (Fig. 3a, green links in circle 1). Furthermore, while the *MIR588;RSPO3* and *LSP1* loci lacked evidence for epistasis by lo-siRF, they were each identified to be marginally associated with LV mass. The specific prioritization order of these loci can be found in Supplementary Table 4, and details regarding the direction or sign of the interactions can be found in Supplementary Note 1. In total, lo-siRF identified 283 SNVs located within the six loci (Extended Data 3, Extended Data Fig. 4). Ninety percent of the 283 SNVs have previously been shown to harbor multiple distinct cardiac function associations^36^ in phenome-wide analyses (e.g., pulse rate, Extended Data 3), suggesting a strong likelihood that these lo-siRF-prioritized loci contribute to determining cardiac structure and function.

### Loci associated with left ventricular mass exhibit regulatory enrichment

We performed functional mapping and annotation (FUMA)^37^ for the 283 lo-siRF-prioritized SNVs (Fig. 1, purple and Fig. 3). For linkage disequilibrium (LD), we used a default threshold of *r*^2^ = 0.6 and chose the UKBB release 2b reference panel created for British and European subjects to match the population group used for lo-siRF prioritization. FUMA identified 572 additional candidate SNVs (Extended Data 4) in strong LD (*r*^2^ > 0.6) with any of the 283 lo-siRF-prioritized SNVs, including 492 SNVs from the input GWAS associations (points in Fig. 3a, circle 8) and 80 non-GWAS-tagged SNVs extracted from the selected reference panel (heatmap tracks in Fig. 3a, circle 8). We then assigned these 572 FUMA-extracted candidate SNVs to a lo-siRF-prioritized locus (Fig. 2f) based on the corresponding lo-siRF-prioritized SNV (out of the 283 SNVs), which has the maximum *r*^*2*^ value with the candidate SNV.

The two loci contributing to the top-ranked epistatic interaction by lo-siRF, the *CCDC141* and *IGF1R* loci (Fig. 2f), both showed a significant enrichment of intronic variants relative to the background reference panel (Fig. 3b, Extended Data 5). Over 88% of the SNVs in or in LD with these two loci were mapped to actively transcribed chromatin states (TxWk) or enhancer states (Enh) in left ventricles based on the ChromHMM Core 15-state model^38^ (Fig. 3a, circle 7). More than 47% and 76% of the identified SNVs in or in LD with the *CCDC141* and *IGF1R* loci, respectively, showed the highest RegulomeDB^37,39^ categorical score (ranked within category 1 from the 7 main categories). The Combined Annotation-Dependent Depletion (CADD) score^40^ was used to judge the deleteriousness of prioritized variants (Extended Data 4). As expected, GTEx^41^ data revealed that 82% of SNVs in or in LD with the *IGF1R* locus are expression quantitative trait loci (eQTLs) for the gene *IGF1R*. In contrast, of the SNVs in or in LD with the *CCDC141* locus, only 14% are eQTLs for gene *CCDC141* and 22% are splicing quantitative trait loci (sQTLs) for gene *FKBP7*. This differential fractionation of eQTLs and sQTLs suggests distinct epistatic mechanisms: the *IGF1R* locus is likely to affect the expression level of gene *IGF1R*, whereas the *CCDC141* locus may play a regulatory role by affecting the alternative splicing of gene *FKBP7*. Furthermore, Hi-C data indicated that all SNVs identified in or in LD with the *IGF1R* locus are in 3D chromatin interaction with gene *SYNM* while more than 54% SNVs identified in or in LD with the *CCDC141* locus are in 3D chromatin interaction with gene *TTN*. These results suggest a possibility of higher-order interactions between more than two genes.

The *CCDC141* and *TTN* loci exhibit genomic proximity (Fig. 3a). Their interaction, however, does not appear to stem from this proximity. Indeed, the *CCDC141* and *TTN* genes have been individually associated with LV mass^42,43^. Due to this proximity, previous studies^44,45^ have assumed *CCDC141* as a secondary gene that affects LV mass through the *TTN* gene expression. However, we found low LD (*r*^*2*^ < 0.6) between any two of the 283 lo-siRF-prioritized SNVs, suggesting an independent role of each locus in their epistasis. In addition, we compared all the epistatic SNVs that were aggregated to the *TTN* locus, including both lo-siRF-prioritized SNVs and their linked variants in LD extracted by FUMA, with their counterparts that were filtered out by lo-siRF. We found that the *TTN* locus showed a significant depletion of SNVs located close to (<10 kb) the gene *CCDC141* (*p* = 2.38E-9, two-sided Fisher exact test). Similarly, the *CCDC141* locus showed a substantially decreased enrichment of SNVs that are close to gene *TTN* (*p* = 0.02, two-sided Fisher exact test). These results suggest that although the *CCDC141* and *TTN* loci are located close to each other in the genome, the prioritized epistatic SNVs are located farther apart relative to randomly selected SNVs from the two loci.

In contrast to the *CCDC141* and *IGF1R* loci, the *TTN* locus showed a significant enrichment of exonic variants and intronic variants that are transcribed into non-coding RNA (ncRNA_intronic, Fig. 3b). Of those exonic variants, 62% are nonsynonymous. This suggests that one mechanism of epistatic effect for the *TTN* locus could be structural alterations in the titin protein. Over 90% of SNVs in or in LD with the *TTN* locus were mapped to actively transcribed states (Tx, TxWk) in left ventricles (Fig. 3a, circle 7). Interestingly, these SNVs were associated with a quiescent chromatin state (Quies) in the right atrium, indicating that the epistatic effects of the *TTN* locus may be specific to ventricular tissues. Nearly half of SNVs in or in LD with the *TTN* locus are eQTLs for the gene *FKBP7*. In addition, 83% of these SNVs are sQTLs for gene *FKBP7* or *TTN*, suggesting a regulatory effect of the *TTN* locus on the expression and splicing of gene *FKBP7*. Moreover, the *TTN* locus was suggested to impact genes *PDE11A*, *RBM45*, *PRKRA*, and *DFNB59* through 3D chromatin interactions.

The hypostatic locus *LOC157273;TNKS* showed a significant enrichment of variants within non-coding RNA regions of exons and introns (Fig. 3b). Over 95% of identified SNVs in or in LD with this locus were mapped to inactive chromatin states (ReprPCWk, Quies) in left ventricles (Fig. 3a, circle 7). This suggests that in the absence of an epistatic partner, the *LOC157273;TNKS* locus is epigenetically quiescent or repressed by polycomb group proteins. In addition, of all the SNVs in or in LD with this locus, 66% are eQTLs for *MFHAS1* or *CLDN23* and 22% are in 3D chromatin interaction with gene *TNKS*.

Functional annotations for the other two lo-siRF-prioritized loci that were marginally associated with LV mass can be found in Extended Data 4 and 5.

### Epistatic loci functionally map to twenty-one protein-coding genes

**Fig. 4:**
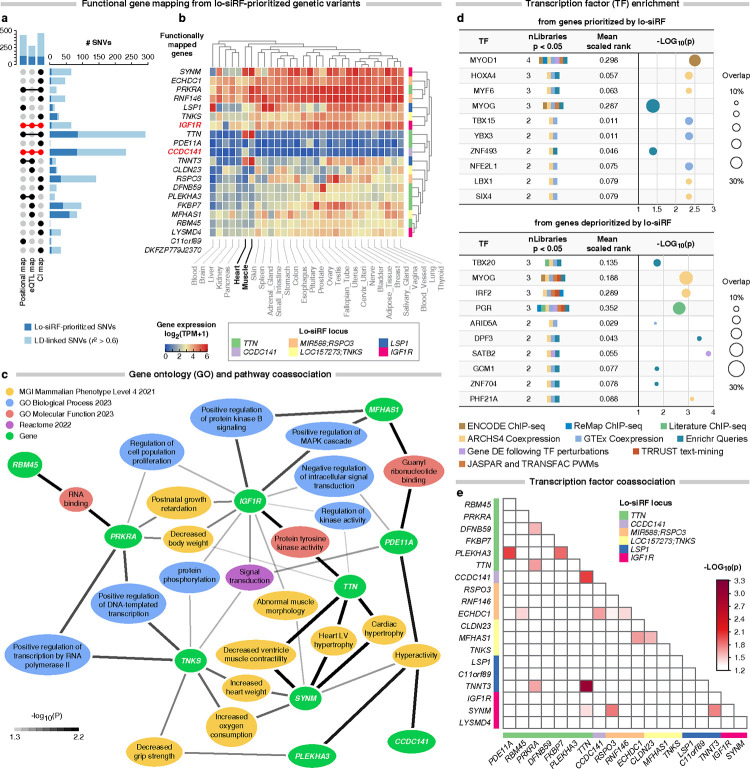
Genes mapped from epistatic loci interact in multiple functional co-association networks. **a**, UpSet plot showing the number of lo-siRF-prioritized SNVs (dark blue) and their LD-linked (*r*^2^ > 0.6) SNVs (light blue, circle 8 in Fig. 3a) that are functionally mapped to each of the 21 protein-coding genes by positional, eQTL, and/or chromatin interaction (CI) mapping using FUMA^37^. *CCDC141* and *IGF1R* (highlighted in red) are prioritized by all the three types of SNV-to-gene mapping. Details can be found in Extended Data 4. **b**, Heatmap of averaged expression (from GTEx) per tissue type per gene (50% winsorization, log_2_(TPM + 1)) for these functionally mapped genes. **c**, Co-association network built from an enrichment analysis integrating multiple annotated gene set libraries for gene ontology (GO) and pathway terms from Enrichr^46^. The co-association network connects top enriched GO and pathway terms with genes (green nodes in the network) functionally linked from lo-siRF-prioritized epistatic and hypostatic loci (Fig. 2f). Strengths (indicated by the edge width in the network) of the co-association between enriched terms and genes were measured and ranked by the empirical *p*-value from an exhaustive permutation of the co-association score for all possible gene-gene combinations in the network (Details in Methods and Extended Data 6). **d,** A comparison between the top 10 transcription factors (TFs) enriched from genes prioritized (top) and deprioritized (bottom) by lo-siRF. The lo-siRF-prioritized genes are genes functionally linked from lo-siRF-prioritized SNVs (panel **a**). The lo-siRF-deprioritized genes are genes functionally linked from SNVs that failed to pass the lo-siRF prioritization thresholds. For each of the two gene groups, enrichment results against nine TF-annotated gene set libraries from ChEA3^47^ and Enrichr^46^ were integrated and ranked by the number of significantly (FET *p* < 0.05) overlapped libraries (numbers in the *nLibraries* column) and the mean scaled rank across all libraries containing that TF (colored boxes in the *nLibraries* column). The balloon plot shows the lowest FET *p*-values for each TF (horizontal axis) and the proportion of overlapped genes (balloon size) between the input gene set and the corresponding TF-annotated gene set. **e**, Heatmap showing the TF co-association strength of gene-gene combinations among lo-siRF-prioritized genes relative to randomly selected gene pairs in the co-association network. More details are available in Extended Data Fig. 5 and Extended Data 7.

Three strategies, positional, eQTL, and chromatin interaction, mapped the six LV hypertrophy risk loci to 21 protein-coding genes (Fig. 4a). Genes prioritized by eQTL and chromatin interaction mapping are not necessarily located in the corresponding risk locus, but they are linked to SNVs within or in LD with the locus (Fig. 3a). Among the 21 genes, *CCDC141* and *IGF1R* were prioritized by all the three mapping strategies (Fig. 4a), suggesting that these two genes are very likely involved in determining LV mass. Interestingly, none of the SNVs mapped to *IGF1R* were statistically significant in our GWAS studies using BOLT-LMM and PLINK (Extended Data Fig. 2 and Extended Data 1). This reveals the potential of lo-siRF to identify risk loci that may be overlooked by GWAS. Based on the expression data from GTEx V8, *TTN*, *TNNT3*, and *SYNM* are up-regulated while *CLDN23* and *MFHAS1* are down-regulated in both heart and muscle tissues (Fig. 4b). In contrast, *CCDC141* is up-regulated specifically in heart tissues whereas *RSPO3* is down-regulated in heart but up-regulated in muscle tissues (Fig. 4b).

### Ten of twenty-one genes mapped from epistatic loci interact in network analysis

We performed gene ontology (GO) and pathway enrichment analysis on the 21 genes mapped from lo-siRF loci. We adopted previously established approaches^46–48^ and integrated enrichment results across libraries from multiple sources to establish a GO and pathway co-association network (Fig. 4c). To evaluate the interaction strength between any two genes in the network, we calculated a co-association score for every possible gene-gene combination (n = 72,771) from both genes prioritized and deprioritized by lo-siRF. Lo-siRF-prioritized genes are the 21 genes functionally mapped from the 283 lo-siRF-prioritized SNVs and their LD-linked SNVs (Fig. 4a). Lo-siRF-deprioritized genes are those functionally mapped from the SNVs that failed to pass the lo-siRF prioritization threshold. Compared to random gene pairs in the network, 10 genes that were functionally mapped from the lo-siRF-prioritized epistatic and hypostatic loci showed significant co-associations with multiple GO/pathways (Fig. 4c, Extended Data 6). Consistent with our hypothesized epistasis (Fig. 2f), gene *CCDC141* showed a significant co-association to *SYNM* (functionally linked to the *IGF1R* locus) and *PDE11A* and *PLEKHA3* (both functionally linked to the *TTN* locus) through the GO term of hyperactivity (excessive movement), which has been linked to increased risk of cardiac disease^49^. Beyond that, *TTN*, *IGF1R*, and *SYNM* are co-associated with kinase activity and cardiac structure related GO terms, indicating that these genes may interactively affect cardiac structure by regulating the process of kinase activity.

### Genes mapped from epistatic loci are co-associated with myogenic regulatory factors

**Extended Data Fig. 5:**
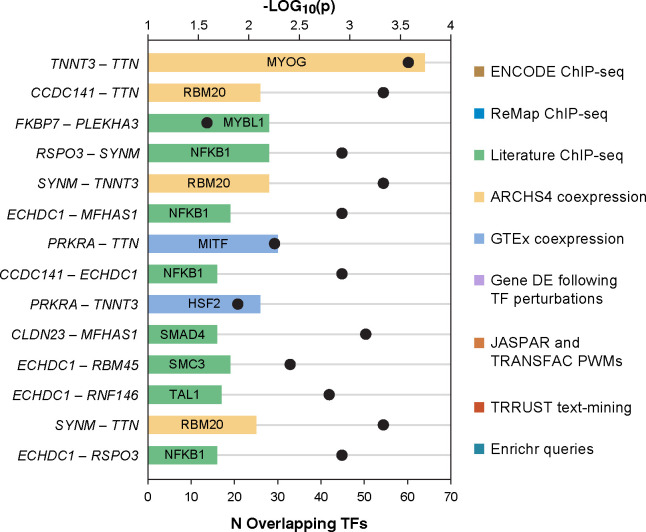
Genes mapped from epistatic loci share transcription factors and splicing regulators. For the gene pairs exhibiting a strong co-association to transcription factors (TFs) and RNA-binding regulators (*p* < 0.05, Fig. 4e), the horizontal bars indicate the number of shared TFs or RNA-binding regulators (bottom axis), of which the top-ranked one with the lowest enrichment *p*-value (dots, top axis) are labeled on the bars, which are colored by the corresponding gene set library. Detailed information can be found in Extended Data 7.

We next performed an integrative enrichment analysis to assess transcriptional regulation of genes prioritized and deprioritized by lo-siRF. Due to assay-specific limitations and biases, we integrated the enrichment results across nine distinct gene set libraries^46,47^ (Fig. 4d, Extended Data 7). We found that the lo-siRF-prioritized epistatic genes shared important myogenic regulatory factors, such as MYOD1, MYF6, and MYOG (Fig. 4d, top). These myogenic regulatory factors coordinate to regulate muscle development and differentiation. In contrast, Transcription factors enriched from lo-siRF-deprioritized genes display a less coordinated regulatory pattern (Fig. 4d, bottom). To further evaluate the interaction strength between any two genes that share transcription factors, we calculated a transcription factor co-association score for all the 72,771 possible gene-gene combinations (see Methods). Compared with random gene pairs,16 gene-gene combinations from the lo-siRF-prioritized genes displayed a significant co-association (empirical *p* < 0.05, Fig. 4e). These co-associations were found in gene-gene combinations from both intra- and inter- lo-siRF-prioritized loci (Fig. 4e). In particular, pairwise combinations among *TTN*, *TNNT3*, *CCDC141*, and *SYNM* share a common splicing regulator, RBM20 (Extended Data Fig. 5). RBM20 has been reported to regulate the alternative splicing of genes important for cardiac sarcomere organization^50^. This suggests that the splicing patterns of these four genes are likely to be co-regulated by RBM20, which is consistent with the exhibited enrichment of sQTLs by the *CCDC141*, *TTN* and *LSP1* lo-siRF loci (Extended Data 4).

### The *CCDC141-TTN* interaction is confirmed in human heart failure transcriptomics

**Fig. 5:**
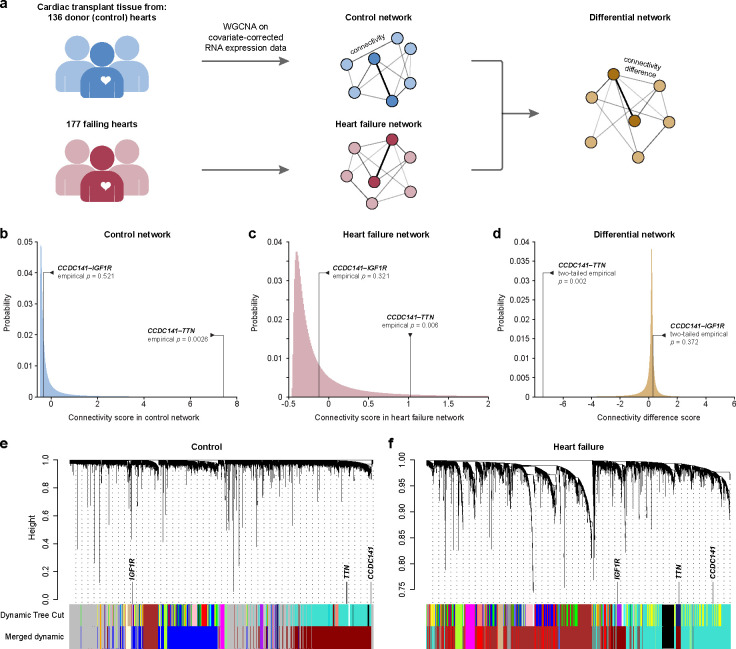
Network analysis using transcriptomic data from 313 human hearts confirms the *CCDC141-TTN* interaction. **a,** Control (blue) and heart failure (red) gene co-expression networks were established from a weighted gene co-expression network analysis (WGCNA) on transcriptomic data obtained from 313 non-failing and failing human heart tissues^51^. **b-c,** The connectivity between lo-siRF-prioritized genes in this study was compared against the full connectivity distributions for all possible gene-gene combinations in the control (**b**) and heart failure (**c**) networks. *CCDC141* and *TTN* show significant connectivity in both networks. **d,** Comparing the difference in connectivities between the control and heart failure networks indicate a change in the connectivity of the hypothesized *CCDC141-TTN* interaction during the progression of failing hearts. **e-f,** Dendrograms from WGCNA control (**g**) and heart failure (**h**) networks show distinctive gene module structures.

We proceeded to the fourth stage for experimental confirmation (Fig. 1, blue) and evaluated how the identified epistases contribute to the progression of heart failure (Fig. 5). We employed a series of weighted gene co-expression networks derived from human cardiac transcriptomic data from 177 failing hearts isolated at the time of heart transplant and 136 non-failing hearts harvested from cardiac transplant donors whose organs were not able to be placed^51^ (Fig. 5a). We compared the molecular connectivity of genes identified as likely epistatic interactors. We defined connectivity as the edge weights between two genes normalized to the distribution of all network edge weights, and compared this to the connectivity of all other available gene-gene combinations in the network. This revealed a strong connection between genes *CCDC141* and *TTN* in the healthy control network (*p* = 0.0026, Fig. 5b). Although these two genes also displayed a significant connectivity (*p* = 0.006, Fig. 5c) in the heart failure network, the connectivity is weakened in the heart failure network (*p* = 0.002, from a third network that measures the connectivity difference between the control and heart failure networks, Fig. 5d). To gain insight into the potential underlying mechanism for this difference, we compared gene module memberships. *CCDC141* and *TTN* are co-associated with the electron transport chain/metabolism module in the control network while *IGF1R* is associated with the unfolded protein response module (Fig. 5e). In the failing hearts, *TTN* and *IGF1R* are co-associated with the muscle contraction/cardiac remodeling module, whereas *CCDC141* remains associated with the metabolism module (Fig. 5f).

### Perturbation confirms epistatic relationships in cardiomyocyte hypertrophy

**Fig. 6:**
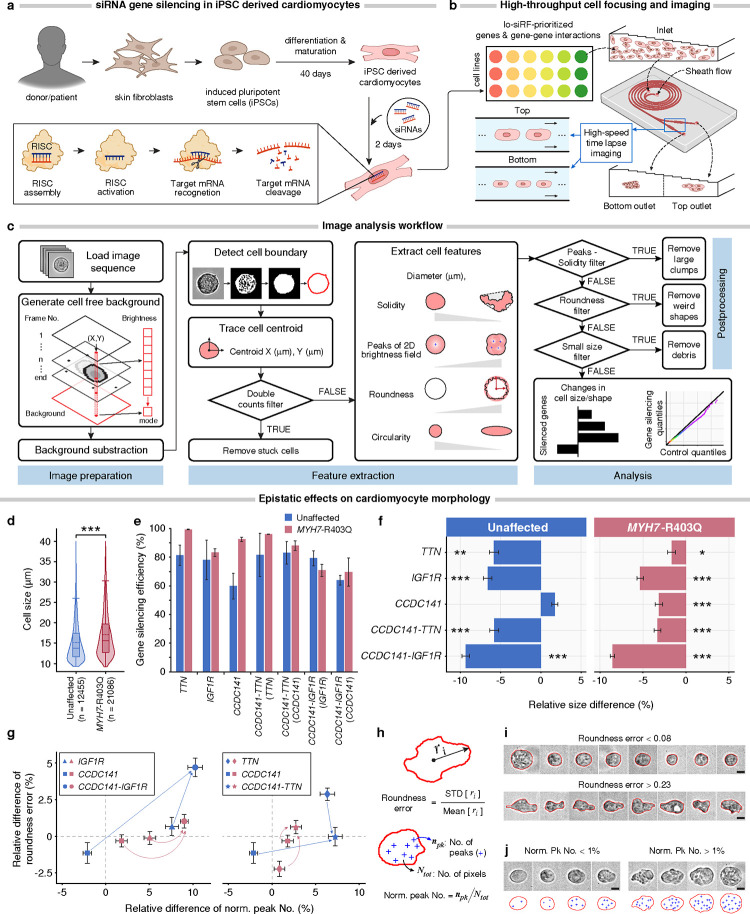
*CCDC141* non-additively interacts with *TTN* and *IGF1R* to modify cardiomyocyte morphology. **a**, Human induced pluripotent stem cell (iPSC)-derived cardiomyocytes with and without hypertrophic cardiomyopathy (carrying an *MYH7*-R403Q mutation) were transfected with scramble siRNA or siRNAs specifically targeting single (*CCDC141*, *IGF1R*, and *TTN*) or combined (*CCDC141*-*IGF1R* and *CCDC141*-*TTN*) genetic loci prioritized by lo-siRF. **b**, Gene-silenced cardiomyocytes were bifurcated into two focused streams of large and small cells using a spiral microfluidic device (cell focusing mechanism illustrated in Extended Data Fig. 6) to allow high-resolution single cell imaging. **c**, Workflow of the image analysis process. Time-lapse image sequences of single cells passing through the top and bottom microchannel outlets (panel **b**) were fed into a customized MATLAB-based program that extracts cell size/shape features via a sequential process of bright field background correction, cell boundary detection, cell tracking and stuck cell removal, cell feature extraction, data quality control and postprocessing, and morphological feature analysis. Extracted single cell features for each gene-silencing condition were compared with their scrambled control values to validate the potential role of epistasis in the genetic regulation of cardiomyocyte hypertrophy (**d-j**). **d**, Violin plots of cell diameters of unaffected (blue) and *MYH7*-R403Q variant (red) cardiomyocytes. Solid and dashed lines in box plots represent median and mean values, respectively. Asterisks indicate significant difference (***p < 1E-36, Wilcoxon signed rank test). **e**, Gene-silencing efficiency in unaffected (blue, n = 5 to 9) and *MYH7*-R403Q variant (red, n = 3) cells based on RT-qPCR analysis (details in Methods). Error bars indicate standard deviations. **f**, Percent change (relative size difference) in median cell diameter of gene-silenced cardiomyocytes relative to scramble control values indicates that *CCDC141* interacts with *IGF1R* to rescue cardiomyocyte hypertrophy. Relative size differences were averaged across data from two to four independent batches of cells. Error bars indicate standard deviations computed on 1000 bootstrap samples of these batches with the following sample size: n = 13147 (*TTN*), 19460 (*IGF1R*), 45304 (*CCDC141*), 19979 (*CCDC141*-*TTN*), and 26135 (*CCDC141*-*IGF1R*) for unaffected cells and n = 22134 (*TTN*), 33801 (*IGF1R*), 21158 (*CCDC141*), 39515 (*CCDC141*-*TTN*), and 52049 (*CCDC141*-*IGF1R*) for *MYH7*-R403Q variant cells. Asterisks indicate significant difference between gene-silencing and scramble control conditions based on the maximum *p*-values of Wilcoxon signed rank test across all batches of cells (*p < 0.05, **p < 0.001, and ***p < 1E-4). **g**, *CCDC141* non-additively interacts with *IGF1R* (left) and *TTN* (right) to modify boundary and texture features of unaffected (blue) and *MYH7*-R403Q variant (red) cells. Cell boundary waveness and texture irregularity were measured by the roundness error (**h**, top) and normalized peak number (**h**, bottom), respectively. **i**, Representative single-cell images overlapped with detected cell boundaries (red lines) show that a higher roundness error indicates increased irregularity of the cell boundary. **j**, Representative single-cell images with detected peaks (blue plus signs) of the brightfield intensity distribution enclosed within the cell boundaries (red lines) indicate a varying level of cell textural irregularity. Scale bars: 10 μm. Detailed statistical information of cell morphology measurements and non-additivity analysis for the studied gene pairs can be found in Extended Data 8.

We interrogated epistatic associations in a genetic model of cardiac hypertrophy (Fig. 1, blue): induced pluripotent stem cell cardiomyocytes derived from patients with and without hypertrophic cardiomyopathy caused by the cardiac myosin heavy chain (*MYH7)* p.R403Q variant^30^ (Fig. 6a). Cardiac myosin heavy chain 7 is a key component of the cardiac sarcomere, and the most common cause of hypertrophic cardiomyopathy^30^. The patient presented with typical symptoms, and echocardiography revealed severe LV hypertrophy and a small LV cavity^30^. At the cellular level, cardiomyocytes exhibit an elevated mean cell size and non-Gaussian size distribution with a long tail relative to the unaffected control (Fig. 6d).

**Extended Data Fig. 6:**
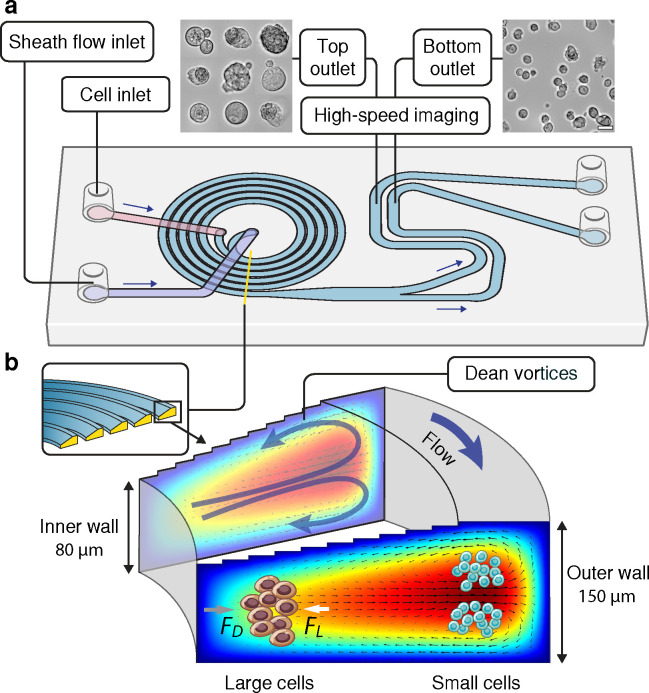
Spiral-shaped inertial microfluidic channel for cell focusing and imaging. **a,** Schematic of an inertial microfluidic cell focusing device. Cell suspensions and fresh medium were introduced into the microfluidic device through the cell and sheath flow inlets, respectively, using a syringe pump and flowed down the 5-loop spiral microchannel with the same flow rate (1.2 mL/min). Inserted microscopy images show that randomly dispersed cells were separated by size and bifurcated into the top (large cells) or bottom (small cells) outlets. Scale bar, 10 μm. Outlet channels are connected to straight observation channels where flowing cells were further focused in the channel height direction and imaged using a high-speed camera for morphological feature extraction (Fig. 6c). **b**, Schematic of the cell focusing principle. The spiral microchannel has a cross-section with a slanted ceiling, resulting in different depths at the inner and outer side of the microchannel. This geometry induces strong Dean vortices (counter rotating vortices in the plane perpendicular to the main flow direction) in the outer half of the microchannel cross-section. The interplay between drag forces $\left(F_{L}\right)$ induced by Dean vortices and lift forces $\left(F_{D}\right)$ due to shear gradient and the channel wall drives cell transverse migration towards equilibrium positions where the net force is zero. As a result, large cells in a heterogeneous population progressively migrate closer to the inner channel wall, while smaller cells move towards the outer channel wall. Details about microchannel dimensions can be found in Methods.

To determine if *CCDC141* can act both independently and in epistatic interactions with other genes to attenuate the pathologic cellular hypertrophy caused by *MYH7*-R403Q, we silenced genes *CCDC141*, *IGF1R*, *TTN*, and gene pairs *CCDC141*-*IGF1R* and *CCDC141*-*TTN* using siRNAs in both diseased and healthy cardiomyocytes and compared them with cells transfected with scramble siRNAs (control) (Fig. 6a and 6e). Phenotypic consequences of these perturbations on cellular morphology were then evaluated in high-throughput using a spiral inertial microfluidic device (Fig. 6b) in combination with automated single-cell image analysis (Fig. 6c). The microfluidic device adopted the Dean flow focusing principle^28^ (details in Extended Data Fig. 6 and Methods) to mitigate the non-uniform cell focusing^52^, thereby enhancing the imaging resolution^53^ affected by the large variations in cardiomyocyte diameter (Fig. 6d).

We first assessed the non-additive effects of the *CCDC141*-*IGF1R* interaction on cardiomyocyte size (Fig. 6f). Bootstrapped hypothesis tests were performed, for which the *p*-values are capped below by *p* < 1E-4 (Extended Data 8). Silencing *IGF1R* alone reduces the median cell size by 5.3% ± 0.4% (*p* < 1E-4) in diseased cells compared to scrambled control and 6.6% ± 0.5% (*p* < 1E-4) in healthy cells. Silencing *CCDC141* alone also decreases median cell size by 3.2% ± 0.5% (*p* < 1E-4) in diseased cells, but had no impact on healthy cells. Digenic silencing of *CCDC141* and *IGF1R* reveals a synergistic effect on attenuating pathologic cell hypertrophy in diseased cells, resulting in an 8.5% ± 0.3% (*p* < 1E-4) decrease in the median cell size. This is consistent in healthy cells, where silencing *CCDC141* alone fails to affect cell size, but digenic silencing of *CCDC141* and *IGF1R* decreases the median cell size by 9.3% ± 0.5% (*p* < 1E-4). Moreover, this effect appears to be non-additive for both healthy and diseased variant cells (*p <* 1E-4 for non-additivity), consistent with an epistatic mechanism. These findings serve to confirm the strongest epistatic association identified by lo-siRF (Fig. 2f).

We found a comparable non-additive effect for the *CCDC141*-*TTN* interaction. Digenic silencing of *CCDC141*-*TTN* leads to a pronounced reduction in median cell size (by 5.8% ± 0.6% for healthy cells and 3.3% ± 0.4% for diseased cells, *p* < 1E-4) relative to monogenic silencing, and appears to be non-additive for both healthy and diseased cells (*p* < 1E-4 for non-additivity). Additionally, *CCDC141* and *TTN* show distinctive independent roles in repressing cardiomyocyte hypertrophy. In healthy cells, monogenic silencing of *TTN* leads to a larger cell size reduction compared to the case of silencing *CCDC141*. In contrast, diseased cells display a larger size reduction in response to monogenic silencing of *CCDC141*.

**Extended Data Fig. 7:**
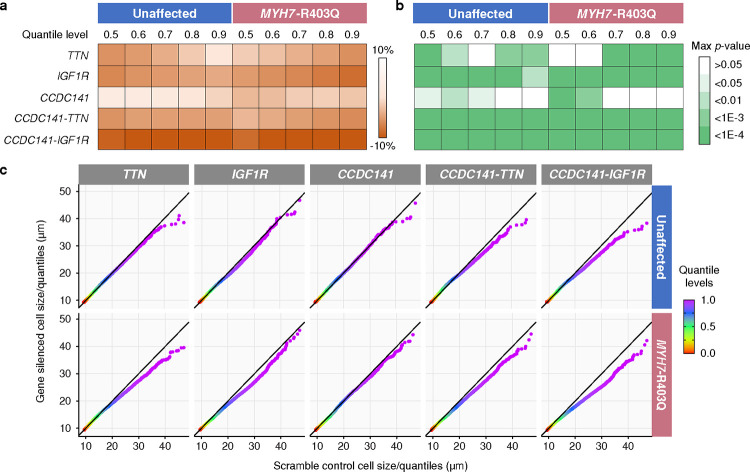
Epistatic genes non-uniformly reshape cardiomyocyte size distributions. **a**, A heatmap of relative differences of cell sizes at various quantile levels between gene-silencing and scramble control conditions for unaffected and *MYH7*-R403Q variant cardiomyocytes. Larger quantiles correspond to larger cells in the cell size distribution. Dark red indicates strong reduction of cell sizes at the specified quantile level in gene-silenced cells relative to the scramble control. The corresponding statistical differences (**b**) were evaluated by the maximum *p*-values across all batches of cells using a bootstrap quantile test (with 10,000 bootstrapped samples). **c**, Representative QQ-plots of cell size quantiles comparing between gene-silenced cells and scramble controls for both unaffected (top row) and *MYH7*-R403Q variant (bottom row) cardiomyocytes indicate a clear size-bias in the effect of silencing *CCDC141*-*IGF1R* on correcting cardiomyocyte hypertrophy.

**Extended Data Fig. 8:**
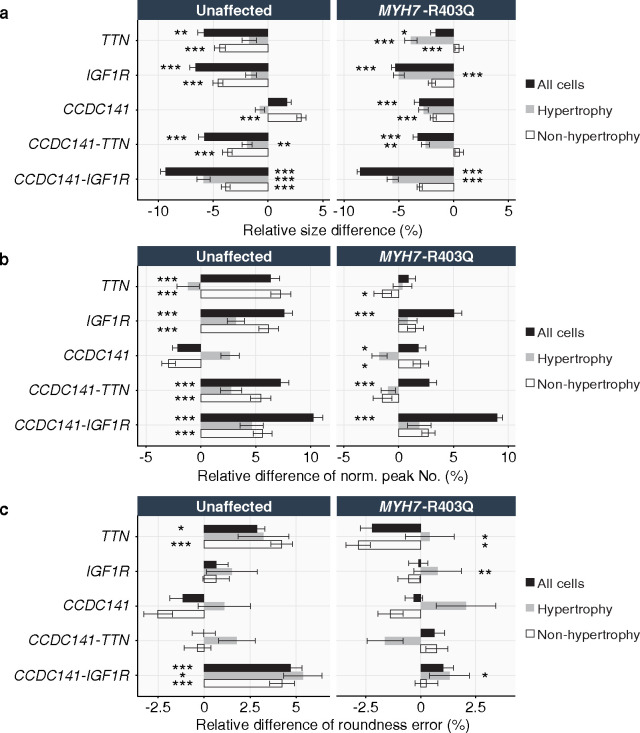
Effects of lo-siRF-prioritized genes and gene-gene interactions on hypertrophic and non-hypertrophic cell morphology. Relative differences in median cell size (**a**), normalized peak number (**b**), and roundness error (**c**) of gene-silenced human induced pluripotent stem cells-derived cardiomyocytes compared to scramble controls computed for hypertrophic cells (size-sorted by microfluidic channel illustrated in Extended Data Fig. 6, gray bars), non-hypertrophic cells (white bars), or both (black bars). Error bars indicate standard deviation calculated from bootstrapping samples of 2 to 4 batches of cells. Asterisks indicate significant differences compared to the scramble control based on the maximum *p*-values of Wilcoxon signed rank test across all batches of cells (*p < 0.05, **p < 0.001, and ***p < 1E-4).

Furthermore, both *CCDC141*-*IGF1R* and *CCDC141*-*TTN* interactions show a stronger effect on rescuing larger cardiomyocytes over smaller ones in both cell lines (Extended Data Fig. 7 and 8). In contrast, monogenic silencing does not exhibit such a non-uniform effect on reshaping the cell size distribution, which reinforces the hypothesized non-additivity of these two epistatic interactions (details in Extended Data 8 and Supplementary Note 2).

Recent studies have shown that cellular morphological features, such as cell boundary and textural irregularities, are informative readouts of cytoskeletal structure, which is highly associated with disease state in hypertrophic cardiomyopathy^29,54^. We analyzed relative changes in cell shape and texture (Fig. 6g) by measuring the counts of peak intensities normalized to the total number of pixels enclosed by the cell boundary (Fig. 6h). Cells with a high normalized peak number display a ruffled texture, which manifests in unevenly distributed 2D intensities (Fig. 6j). Our analysis shows that silencing both *CCDC141* and *IGF1R* (circles in Fig. 6g, left) yields a larger increase in intensity peak number than silencing *IGF1R* alone (triangles in Fig. 6g, left) for both cell lines, exhibiting a synergistic epistasis between *CCDC141* and *IGF1R* (*p* < 1E-4 for non-additivity). We also analyzed cell roundness error, a measure of how far radii measured on the cell outline deviate from a perfect circle (Fig. 6h). This parameter increases with an increasing cell boundary waviness or elongation (Fig. 6i). We show that the silencing of *CCDC141* and *IGF1R* synergistically interact to increase roundness error of diseased cardiomyocytes (*p* < 1E-4 for non-additivity, Fig. 6g, left). In addition, *CCDC141* and *TTN* display antagonistic epistasis and synergistic epistasis in their impact on roundness error for healthy and diseased cells (*p* < 1E-4 for non-additivity, Fig. 6g, right), respectively.

## Discussion

While computational models^17,18^ have supported epistatic contributions to human complex traits and disease risk, examples in the literature are rare, with even fewer experimentally confirmed. Here, we developed a veridical machine learning^23^ approach to identify epistatic associations with cardiac hypertrophy derived from a deep learning model that estimates LV mass from cardiac imaging of almost thirty thousand individuals in the UK Biobank. We report novel epistatic effects on LV mass of common genetic variants associated with *CCDC141*, *TTN*, and *IGF1R.* We used established tools to functionally link risk loci to genes, and then confirmed gene level co-associations through network analyses, including via shared transcription factors and pathways enriched against multiple annotated gene set libraries and co-expression networks we built using transcriptomic data from over three hundred healthy and diseased human hearts. Finally, using a cellular disease model incorporating monogenic and digenic silencing of individual genes, we assessed phenotypic changes in cardiomyocyte size and morphology using a novel microfluidic system, confirming the non-additive nature of the interactions.

Our approach advances epistasis discovery in several key ways. First, unlike studies relying on linear-based models^55–58^, we leverage a more realistic, nonlinear tree-based model that mirrors the thresholding (or switch-like) behavior commonly observed in biomolecular interactions^59^. Second, in contrast to other tree-based approaches that evaluate interactions on a variant-by-variant basis^60–64^, our novel stability-driven importance score consolidates individual variants into loci for the assessment of feature importance, allowing for more reliable extraction of epistatic interactions from weak association signals. This is particularly valuable for evaluating non-coding variants. Moreover, instead of exhaustively searching all possible interactions, signed iterative random forests internally employ a computationally-efficient algorithm, which automatically narrows the search space of interactions to only those that stably appear in the forest and thus achieves a scalability much higher than existing tree-based approaches^62,65^. This allows lo-siRF to handle larger datasets without the need for LD pruning before the interaction search, which may inadvertently eliminate important epistatic variants, given that epistasis between loci in strong LD has been evidenced by a recent study^66^. Furthermore, our computational prioritization is rigorously validated through multiple functional network analyses and robust experimental confirmation.

Our results add to a small literature on epistasis in cardiovascular disease. Two recent studies have found epistasis influencing the risk of coronary artery disease^17,18^. Li et al.^18^ identified epistasis between *ANRIL* and *TMEM106B* in coronary artery tissues. Although their method predicted functionally interpretable interactions between risk loci of interest, they relied heavily on prior knowledge and careful selection of the causal gene pairs,^18^ making the approach challenging to scale. Zeng et al.^17^ used population-scale data and performed epistasis scans from regions around 56 known risk loci. This study identified epistasis between variants in *cis* at the *LPA* locus without experimental confirmation. In contrast, our approach allows discovery of not only cis-epistasis, but also long-range interactions between interchromosomal loci (e.g., *CCDC141* and *IGF1R*) and is supported by gene perturbation experiments. More importantly, both previous studies searched for interactions around known risk loci identified by genome-wide association, which can be far away from the possible epistatic or hypostatic loci that are statistically insignificant in linear univariate association studies. In addition, both studies relied on a logistic regression model, which imposes restrictive assumptions that can be avoided using a nonlinear machine learning approach as in lo-siRF.

Our study has limitations. We focused this analysis on a single ancestry in order to enhance the likelihood of finding reliable interactions from weak association signals. These findings cannot be automatically applied to others. It was not feasible to conduct a formal genetic replication study because the UK Biobank is the only large-scale population cohort with integrated cardiac magnetic resonance images and genetic data. However, to help reduce the possibility of overfitting and increase generalizability, lo-siRF employed numerous stability analyses (see Supplementary Note 1) in addition to a proper training-validation-test data split. Beyond these computational checks, we also present functional supporting evidence and experimental validation. Our computational prioritization via lo-siRF currently groups SNVs based on genomic proximity, without accounting for their functional interdependencies, but this could be addressed by integrating functional annotation into the lo-siRF pipeline. Lastly, lo-siRF is not as scalable as linear-based methods, though it is more scalable than alternative tree-based methods for epistasis detection. It also should be noted that although this study did not identify stable higher-order (> order-2) interactions due to the weak association signal between SNVs and LV mass, the method exhibits the capability to detect such interactions for broader phenotypes and complex traits without incurring additional computational cost.

In summary, our work adds to the discovery toolkit for the genomic architecture of complex traits and expands the scope of genetic regulation of cardiac structure to epistasis.

## Online Methods

### Study participants

The UK Biobank (UKBB) is a biomedical database with detailed phenotypic and genetic data from over half a million UK individuals between ages 40 and 69 years at recruitment^67^. In this study, we restricted our analysis to the largest ancestry subset (i.e., the White British population) of 29,661 unrelated individuals who have both genetic and cardiac magnetic resonance imaging (MRI) data from the UKBB (Supplementary Table 1). More specifically, we considered only those individuals from the UKBB cohort who self-reported as White British and have similar genotypic backgrounds based on principal components analysis as described in prior work^67^. We also identified related individuals (i.e., third-degree relatives or closer) via genotyping and omitted all but one individual from each related group in the analysis. Details regarding this cohort refinement have been described and implemented previously^67,68^. This refinement resulted in a cohort of 337,535 unrelated White British individuals from the UKBB, of which 29,661 have both genetic and cardiac MRI data. We randomly split this data into training, validation, and test sets of size 15,000, 5,000, and 9,661 individuals, respectively.

### Genotyping and quality control

For the study cohort of 29,661 individuals described above, we leveraged genotype data from approximately 15 million imputed autosomal SNVs. These have been imputed from 805,426 directly assayed SNVs (obtained by the UKBB from one of two similar Affymetrix arrays) using the Haplotype Reference Consortium and UK10K reference panels^67^. Imputed variants were subject to several quality-control filters, including outlier-based filtration on effects due to batch, plate, sex, array, and discordance across control replicates. Further, we excluded variants due to extreme heterozygosity, missingness, minor allele frequency (< 10^−4^), Hardy-Weinburg equilibrium (< 10^−10^), and poor imputation quality (< 0.9). Further details can be found in previous studies^67,68^.

### Quantification of left ventricular hypertrophy

We retrieved cardiac MRI images from 44,503 UKBB participants, taken during their most recent imaging visit, and closely followed the method previously described by Bai et al.^25^. A fully convolutional network^25^ was previously trained using a dataset of 4,875 subjects with 93,500 pixelwise segmentations of UKBB short-axis cardiac MRI multi-slice images generated manually with quality control checks for inter-operator consistency^69^. The network architecture employed multiple convolutional layers to learn image features across five resolution scales. Each scale involved two or three convolutions followed by batch normalization and ReLU transformation. Feature maps from the five scales were upsampled back to the original resolution, combined into a multi-scale feature map, and processed through three additional convolutional layers, followed by a softmax function to predict the segmentation label for each pixel. This approach generates a pixelwise segmentation map for each MRI slice which was previously determined to be at a level of accuracy similar to human experts^25^. We applied this trained deep learning model to our entire dataset of 44,503 cardiac MRIs. This resulted in segmentations of the LV cavity and myocardium from each short axis frame, which allowed for both an area calculation of each segment as well as the application of quality control checks^25^ based on consistency within and between slices and time steps. There were 44,219 segmentations that passed the quality control. Using the calculated areas, we computed the volume of the LV myocardium through simple integration over slices. This volume was then converted to a left ventricular mass (LVM) using a standard density estimate of 1.05 g/mL^32^. LVMi was computed by dividing LVM by an estimate of body surface area based on height and body weight calculated using the Du Bois formula^33^. From the 44,219 segmentations, we restricted the analysis to LVMi measurements for 29,661 unrelated White British individuals using the measurements from their most recent imaging visit if multiple imaging visits were recorded.

### Lo-siRF step 1: Dimension reduction of variants via genome-wide association studies

As the first step in the lo-siRF pipeline, we performed a genome-wide association study (GWAS) on the training data for the rank-based inverse normal-transformed LVMi using two algorithms, PLINK^34^ and BOLT-LMM^35^, in order to filter the number of features from over 15 million SNVs to a more computationally-feasible size (Fig. 2b). This step is akin to typical screening phases in fine-mapping^70^ and other tree-based epistasis detection methods^63,71^. Since BOLT-LMM and PLINK rely on different statistical models, we chose to employ both implementations to mitigate the dependence of downstream conclusions on this arbitrary choice. Specifically, for the first GWAS run, we fitted a linear regression model, implemented via ‘glm’ in PLINK^72^. For the second GWAS run, we used BOLT-LMM^35^, a fast Bayesian-based linear mixed model method. Each GWAS was adjusted for the first five principal components of ancestry, gender, age, height, and body weight. We then ranked the SNVs by significance (i.e., the GWAS *p*-value) for each GWAS run separately and took the union of the top 1000 SNVs (without clumping) from each of the two GWAS runs. This resulted in a set of 1405 GWAS-filtered SNVs that were used in the remainder of the lo-siRF pipeline. Here, we chose to use the top 1000 SNVs per GWAS method (without clumping) as it yielded the highest validation prediction accuracy compared to choosing other possible thresholds (500 and 2000 SNVs per GWAS with and without clumping). Though the GWAS is not the focus of this work, we provide a summary of the PLINK and BOLT-LMM GWAS results for completeness and for comparison in Extended Data 1. We also provide a list of the 1405 GWAS-filtered SNVs in Extended Data 2. We note that these 1405 GWAS-filtered SNVs strictly contain the SNVs that passed the genome-wide significance threshold (*p* = 5E-8).

### Lo-siRF step 2: Binarization of the left ventricular mass phenotype

Next, we binarized the raw (continuous) LVMi phenotype into a low and a high LVMi group before fitting signed iterative random forest (Fig. 2c). That is, for a given threshold *x*, we binned individuals within the top and bottom *x*% of LVMi values into two classes with the high and low LVMi values, respectively, while omitting the individuals in the middle quantile range. Due to the sex-specific biological variation of LVMi (Supplementary Note 1), we performed this binarization for males and females separately. For males, low and high LVMi was considered under 43.8–46.0 g/m^2^ and above 55.4–58.5 g/m^2^, respectively, depending on our choice of binarization threshold (Supplementary Table 2). For females, low and high LVMi was defined as under 35.1–36.8 g/m^2^ and above 43.8–46.1 g/m^2^, respectively, depending on our choice of binarization threshold. We performed this binarization step in order to simplify the original low-signal regression problem into a relatively easier classification task: to predict whether an individual has a very high or very low LVMi, in essence, denoising the LVMi signal in the data. Moreover, as we will see in the next section, this binarization helped us more readily interpret and assess the performance of prediction methods with respect to the prediction screening step of the PCS framework^23^. Since the specific threshold choice is arbitrary, we ran the remainder of the lo-siRF pipeline using three different binarization thresholds (15%, 20%, 25%) that balance the amount of denoising and data lost. In the end, we aggregated the results that were stable across all three binarization thresholds, described in the Method section Lo-siRF step 4.4: Ranking genetic loci and interactions between loci.

### Lo-siRF step 3: Prediction

#### Lo-siRF step 3.1: Fitting signed iterative random forest on the binarized LV mass index phenotype

For each binarization threshold, we trained a signed iterative random forest (siRF) model^20^ using the 1405 GWAS-filtered SNVs to predict the binarized LVMi phenotype and generate candidate interactions for further investigation (Fig. 2d). siRF first iteratively grows a sequence of feature-weighted random forests, re-weighting features in each iteration proportional to their feature importance from the previous iteration in order to stabilize the decision paths. Then, provided that the resulting stabilized forest provides reasonable prediction performance (see the Methods section Lo-siRF step 3.2: Prediction check), siRF leverages a computationally-efficient algorithm, random intersection trees^73^, to identify nonlinear higher-order interaction candidates based on frequently co-occurring features on a decision path. Intuitively, sets of features that frequently co-occur along a decision path together are more likely to interact and are identified by siRF. siRF is particularly attractive for prioritizing epistatic interactions as (1) it offers an interaction search engine that can automatically search for higher-order interactions with the same order of computational cost as a traditional random forest, and (2) the thresholding behavior of its decision trees resembles the thresholding (or switch-like) behavior commonly observed in biomolecular interactions^59^. Further, siRF improves upon its predecessor, iterative random forests^19^, by not only tracking which sets of features commonly co-occur on decision paths, but also the sign of the features, i.e., whether low values (denoted *X*^−^) or high values (denoted *X*^*+*^) of feature *X*, appear on the decision path. We refer to Kumbier et al.^20^ for details, but in brief, the signed feature *X*^−^ (or respectively, *X*^*+*^) signifies that a decision rule of the form *X* < *t* (or respectively, *X > t*) for some threshold *t* appeared on the decision path. siRF hence outputs a list of candidate *signed* interactions, where each signed interaction consists of two or more signed features that frequently co-occur on the same decision path. Note when applying siRF to SNV data in practice, the signed feature *SNV*^*+*^ typically represents a heterozygous or homozygous mutation while the signed feature *SNV*^−^ typically represents no mutation at the locus. The following hyperparameters were used to train siRF using the iRF2.0 R package: number of iterations = 3, number of trees = 500, number of bootstrap replicates = 50, depth of random intersection tree (RIT) = 3, number of RIT = 500, number of children in RIT = 5, and minimum node size in RIT = 1. We fit siRF using 10,000 training samples (randomly sampled out of the 15,000 total training samples) and reserved the remaining 5,000 training samples for selecting genetic loci for the permutation test (see the Method section Lo-siRF step 4.3: Permutation test for difference in local stability importance scores).

#### Lo-siRF step 3.2: Prediction check

Per the PCS framework for veridical data science^23^, we next assessed the validation prediction accuracy of siRF (Fig. 2d) to evaluate whether the learnt model is capturing some biologically-relevant phenotypic signal, rather than simply noise, before proceeding to interpret this model in step 4 of lo-siRF. To serve as baseline comparisons, we fit other popular prediction methods, namely, *L*_*1*_-regularized (LASSO) logistic regression^74^, *L*_*2*_-regularized (ridge) logistic regression^75^, random forests^76^, and support vector machines^77^, and we evaluated prediction performance according to multiple metrics, namely, classification accuracy, area under the receiver operator curve, and area under the precision-recall curve. We observed that the prediction power of siRF, though weak (~55% balanced classification accuracy), was greater than these other commonly used prediction methods across all binarization thresholds and evaluation metrics, except for the 15% binarization threshold where siRF performed second-best with respect to classification accuracy (Supplementary Table 3). Since siRF performed better than random guessing (i.e., >50% balanced classification accuracy, which is not guaranteed given the high phenotypic diversity of the LVMi trait) and demonstrated higher prediction power than alternative popular prediction methods, we deemed that the siRF fit for LVMi passed the prediction screening step of the PCS framework. Hence, we proceeded to interpret this siRF model and prioritize candidate interactions in step 4 of lo-siRF. We note also that this prediction check played a key role in our choice of phenotypic data. Prior to studying LVMi, we attempted to run a similar analysis to predict hypertrophic cardiomyopathy (HCM) diagnosis, defined as any ICD10 billing code diagnosis of I42.1 or I42.2 in the UKBB data. However, neither siRF nor the other aforementioned prediction methods passed the 50% balanced classification accuracy requirement for predicting HCM diagnosis. We thus chose not to proceed with the HCM analysis given the poor prediction accuracy and uncertain relevance between the prediction models and the underlying biological processes. This failed prediction check motivated the need for a more refined phenotypic measure of cardiac hypertrophy, which ultimately led to the deep learning extraction of cardiac MRI-derived LVMi. Further discussion of the HCM analysis can be found in Supplementary Note 1.

### Lo-siRF step 4: Prioritization

To lastly interpret the siRF fit for LVMi, we developed a novel stability-driven importance score to prioritize genetic loci and more interestingly, interactions between loci for follow-up experimental validation (Fig. 2e). The assessment of importance at the level of genetic loci, rather than for individual variants, is necessary since variant-level importances here are incredibly unstable (detailed in Supplementary Note 1). This is due to the high correlation between SNVs in LD and the weak phenotypic signal. Consequently, our new importance score aims to aggregate weak, unstable variant-level importances into stronger, more stable locus-level importances by (1) assigning each variant to a genetic locus, (2) evaluating the local (per-individual) importance of each genetic loci or interaction between loci from the siRF fit via a stability-driven measure, and (3) conducting a permutation test to summarize the importance of the genetic locus or interaction between loci across all individuals. We provide details for each step next.

#### Lo-siRF step 4.1: Aggregation of SNVs into loci

We aggregated SNVs into a genetic locus based on genomic proximity. Specifically, we used ANNOVAR^78^ to assign each SNV that appears in the siRF fit to a genetic locus according to the hg19 refSeq Gene annotations (i.e., given by the ‘Gene.refGene’ column in the ANNOVAR output). ANNOVAR uses a default of 1 kb as the maximum distance between SNVs and gene boundaries. Note that from these annotations, each SNV is assigned to exactly one genetic locus. Thus, herein in the context of lo-siRF, a genetic locus is a (non-overlapping) group of SNVs, and a signed genetic locus is a (non-overlapping) group of signed SNVs with the specified sign (i.e., *Locus*^*+*^ consists of ${SNV}_{1}^{+},\;\ldots ,\;{SNV}_{p}^{+}$ while *Locus*^*−*^ consists of ${SNV}_{1}^{-},\;\ldots ,\;\left.{SNV}_{p}^{-}\right)$.

#### Lo-siRF step 4.2: Local stability importance score

We next measured the importance of a genetic locus or interaction between loci based on their stability, or frequency of occurrence, within the siRF fit. However, because the number of variants assigned to each genetic locus can vary, the raw frequency of occurrence will be biased towards larger loci (i.e., those with more variants). A more detailed discussion is provided in Supplementary Note 1. To address this issue, we developed a *local* (or per-individual) *stability importance score*, which quantifies the importance of a signed locus or interaction between loci for making the prediction for each individual. Let $G=\left\{g_{1},\ldots ,g_{K}\right\}$ denote a signed order-𝐾 interaction involving the signed genetic loci $g_{1},\;\ldots \;g_{K}$, and let $v_{1}^{\left(j\right)},\;\ldots ,\;v_{p_{j}}^{\left(j\right)}$ denote the signed SNVs belonging to the signed genetic locus $g_{j}$. Then given a forest T, a signed interaction between loci G, and individual i, the *local stability importance score*, ${LSI}_{T}(G,i)$, is defined as $D_{T}(G,i)/|T|$, where |T| is the number of trees in the forest T, and $D_{T}(G,i)$ is the number of decision paths in the forest T for which two criteria are satisfied: (1) individual i appears in its terminal node and (2) for each j=1,...,K, there exists an $l\;\in \;\{1,\;.\;.\;.\;,\;p_{j}\}$ such that $v_{l}^{\left(j\right)}$ was used in a decision split along the path (Supplementary Data Fig. 3a). In other words, $LSI_{T}(G,i)$ is the proportion of trees in the forest T for which at least one signed variant from each signed locus in the signed interaction G was used in making the prediction for individual i. A higher score indicates greater importance of the signed interaction G for individual i’s prediction. Note that a genetic locus can be viewed as an order-1 interaction, and thus, this local stability importance score can also be computed to assess the (marginal) importance of a single genetic locus.

#### Lo-siRF step 4.3: Permutation test for difference in local stability importance scores

Once we obtained these local stability importance scores for each individual, we performed a two-sample permutation test to assess whether the local stability importance scores for a given signed locus or interaction between loci, G, are different between individuals with high and low LVMi (conditional on the rest of the fitted forest). More formally, the proposed permutation test tests the null hypothesis L=H versus the alternative hypothesis L≠H, where L and H are the distributions of local stability importance scores for individuals with low and high LVMi, respectively. If the local stability importance scores are indeed different between high and low LVMi individuals, the permutation test results in a small *p*-value, indicating that G can differentiate between individuals with high versus low LVMi and hence is an important locus or interaction between loci for LVMi. We performed this permutation test using 10,000 permutations, the difference in means as the test statistic, and the 5,000 validation samples. To bolster the reliability of our findings, we only tested a conservative subset of genetic loci and interactions between loci that passed predictive and stability checks in accordance with the PCS framework. Namely, we tested:

- The top 25 genetic loci, ranked by their average local stability importance scores across 5,000 samples. These 5,000 samples were previously set-aside from within the 15,000 training samples and were not used in fitting the siRF (see the Methods section Lo-siRF step 3.1: Fitting signed iterative random forest on the binarized LV mass index phenotype);
- The signed interactions between loci that were stably identified by siRF across 50 bootstrap replicates. Here, we performed the random intersection trees search within siRF at the locus-level (i.e., using the variant-to-locus assignment as the hyper-features or ‘varnames.grp’ argument when running siRF in R), and we defined a “stable” interaction as one that passed the following siRF stability metric thresholds: stability score > 0.5, stability score for mean increase in precision > 0, and stability score for independence of feature selection > 0^19,20^ (Supplementary Table 5). Details on the siRF interaction and stability metrics can be found in previous work^20^.

#### Lo-siRF step 4.4: Ranking genetic loci and interactions between loci

To select the top lo-siRF recommendations for follow-up experimental validation, we incorporated one final stability check, recommending only those signed loci and interactions between loci that underwent the permutation test and yielded a *p*-value < 0.1 in all three binarization runs. For these signed loci and interactions between loci that were stably important in all three binarization runs, we ranked them by the mean permutation *p*-value, averaged across the three binarization thresholds (Supplementary Table 4). Because of our emphasis on prioritizing candidates for experimental validation, if both the + and − version of the signed locus (or interaction) appear, the final prioritized loci (or interaction) are ranked according to the smaller one of the two *p*-values (Fig. 2f). We note that though the signed information is not pertinent to our goal of recommending candidates for experiments, the signed information from siRF provides more granular information that can improve our interpretation of the fit, and we discuss this further in Supplementary Note 1. We also provide the permutation *p*-values for all conducted permutation tests (including the loci and interactions between loci that were unstable across binarization thresholds) in Supplementary Note 1.

### lo-siRF: PCS documentation and additional stability analyses

In an effort to facilitate transparency of human judgment calls that are inevitably made here and throughout our veridical machine learning pipeline, we provide extensive documentation and discussion of these human judgment calls in Supplementary Note 1. In particular, Supplementary Note 1 includes a discussion of our reasoning and motivation behind the choice of phenotypic data as well as choices in the dimension reduction, binarization, prediction, and prioritization steps. We also performed additional stability analyses in accordance with the PCS framework^23^, detailed in Supplementary Note 1, to ensure that our findings are stable and robust to these human judgment calls (e.g., the choice of GWAS method and binarization threshold) and to bolster the reproducibility of our findings. Supplementary Note 1 is an HTML document, which can be downloaded and displayed in a browser or found at https://yu-group.github.io/epistasis-cardiac-hypertrophy/.

### Functional interpretation of lo-siRF-prioritized variants

#### Functional interpretation step 1: Extraction of candidate SNVs and LD structures

Our lo-siRF approach described above identified a total of 283 SNVs located within 6 LVMi genetic risk loci (Fig. 2f). In order to explore the functional consequences of these prioritized genetic variants and identify genes that are potentially involved in the trait of LV hypertrophy, we performed functional mapping and annotation using a web-based platform, FUMA (v1.5.4)^37^. The SNP2GENE function in FUMA was used to incorporate LD structure and prioritize candidate genes. Taking the GWAS summary statistics from PLINK^34^ and BOLT-LMM^35^ as an input, we submitted the 283 lo-siRF-prioritized SNVs into SNP2GENE as predefined SNVs. This allows SNP2GENE to define LD blocks for each of the 283 lo-siRF-prioritized SNV and use the given 283 SNVs and SNVs in LD with them for further annotations. We adopted the default *r*^2^ threshold (i.e., 0.6) for defining independent significant SNVs. Because any two of the 283 lo-siRF-prioritized SNVs are in LD with each other at *r*^2^ < 0.6, all of the 283 SNVs were defined as independent significant SNVs by FUMA. In order to match the population group used for our lo-siRF prioritization, the reference panel from UKBB release 2b that FUMA created for British and European subjects was chosen for the computation of *r*^2^ and minor allele frequencies. A total of 572 candidate SNVs in strong LD (*r*^2^ < 0.6) with any of the 283 independent significant SNVs were extracted from both the inputted GWAS (with the maximum *p*-value threshold being 0.05) and the reference panel. These 572 candidate SNVs were then assigned to one of the six lo-siRF-identified locus (Fig. 2f) based on its corresponding independent significant SNV, which showed the maximum *r*^*2*^ value in LD with the given candidate SNV. A combination of the 283 independent significant SNVs and the 572 FUMA-extracted candidate SNVs in LD with the independent significant SNVs (details in Extended Data 4) was defined as the lo-siRF-prioritized SNV set, which was used to generate the list of lo-siRF-prioritized genes (Fig. 4a) for the following enrichment analysis (Fig. 4c–4e). As a comparison to the lo-siRF-prioritized SNV set, we uploaded all 1405 GWAS-filtered SNVs (Extended Data 2) as the predefined SNVs in a separate SNP2GENE job. Using the same approach and parameter settings, 929 independent significant SNVs were identified within the given 1405 GWAS-filtered SNVs, and 5771 candidate SNVs in LD with the 929 independent significant SNVs were extracted by FUMA. A combination of the 929 independent significant SNVs and the 5771 candidate SNVs were defined as the reference SNV set. This reference SNV set is purely generated from GWAS prioritization and excludes the evaluation of epistatic effects between genetic variants by lo-siRF. Genes functionally mapped from the reference SNV set was used as a comparison group for the lo-siRF-prioritized gene list in the following enrichment analysis to explore the specific contribution of the identified epistatic genes in the enriched gene ontologies, pathways, and transcription factors (Fig. 4c–4e).

#### Functional interpretation step 2: ANNOVAR enrichment test

To evaluate the functional consequences of the lo-siRF-prioritized genetic loci, we performed ANNOVAR enrichment test of the aforementioned 283 independent significant SNVs and 572 candidate SNVs in LD with them against the selected reference panel in FUMA. The FUMA SNP2GENE process generated unique ANNOVAR^78^ annotations for all the identified SNVs. The enrichment score for a given annotation in a given lo-siRF-prioritized genetic locus (Fig. 3b) was computed as the proportion of SNVs associated to that locus with the given annotation divided by the proportion of SNVs with the same annotation relative to all available SNVs in the reference panel. For the *i*^*th*^ ANNOVAR annotation in the *j*^*th*^ lo-siRF-prioritized locus, the enrichment *p*-value was computed by performing a two-sided Fisher’s exact test on the 2-by-2 contingency table containing $n_{j}(i)$, $\sum_{t}n_{j}\left(t\right)-n_{j}\left(i\right),\;N\left(i\right)-n_{j}\left(i\right)$, and $\sum_{t}N\left(t\right)\;-\sum_{t}n_{j}\left(t\right)-n_{j}\left(i\right)$. Here, $n_{j}(i)$ is the number of SNVs with the i^*th*^ annotation in the j^*th*^ lo-siRF-prioritized locus, N(i) is the number of SNVs with the i^*th*^ annotation in the reference panel, $\sum_{t}n_{j}\left(t\right)$ is the summation of $n_{j}(i)$ for all available annotations in the j^*th*^ lo-siRF-prioritized locus, and $\sum_{t}N\left(t\right)$ is the summation of N(i) for all available annotations in the reference panel. Detailed information can be found in Extended Data 5.

#### Functional interpretation step 3: Functional annotations

In addition to ANNOVAR annotations, FUMA annotated all 283 independent significant SNVs and 572 SNVs in LD with them for functional consequences on potential regulatory functions (core-15 chromatin state prediction and RegulomeDB score) and deleterious effects (CADD score). In particular, the core-15 chromatin state was annotated to all SNVs of interest by ChromHMM^38^ derived from 5 chromatin markers (H3K4me3, H3K4me1, H3K36me3, H3K27me3, and H3K9me3) for 127 tissue/cell types, of which left ventricle (E095), right ventricle (E105), right atrium (E104), and fetal heart (E083) were taken into consideration in this study (Fig. 3a, circle 7). Data and a corresponding description of the core-15 chromatin state model can be found at https://egg2.wustl.edu/roadmap/web_portal/chr_state_learning.html. RegulomeDB^37,39^ annotations guide interpretation of regulatory variants through a seven-level categorical score, of which the category 1 (including 6 subcategories ranging from 1a to 1f) indicates the strongest evidence for a variant to result in a functional consequence. Because the RegulomeDB database (v1.1) used in FUMA has not been updated, we queried all SNVs identified by lo-siRF and FUMA in the RegulomeDB database v2.2 (https://regulomedb.org/regulome-search). Annotations for deleteriousness were obtained from the CADD database (v1.4)^40^ by matching chromosome, position, reference, and alternative alleles of all SNVs. High CADD scores indicate highly deleterious effects of a given variant. A minimum threshold CADD score of 12.37 was suggested by Kircher et al.^40^. In addition to the aforementioned functional annotations, we extracted information of eQTLs and sQTLs for all independent significant SNVs and SNVs that are in LD with one of the independent significant SNVs from GTEx v8^41^. The eQTL information was used for eQTL gene mapping as described in the following section.

#### Functional interpretation step 4: Functional gene mapping

In SNP2GENE, we performed three functional gene mapping strategies – positional, eQTL, and 3D chromatin interaction mapping – using the lo-siRF-prioritized SNV set and the reference SNV set described in the Methods section Functional interpretation step 1: Extraction of candidate SNVs and LD structures. For positional mapping^37,39^, a default value of 10 kb was used as the maximum distance between SNVs and genes. For eQTL mapping, cis-eQTL information of heart left ventricle, heart atrial appendage, and muscle skeletal tissue types from GTEx v8^41^ was used. Only significant SNV-gene pairs (FDR < 0.05 and *p* < 1E-3) were used for eQTL mapping. For 3D chromatin interaction mapping, Hi-C data of left ventricle tissue from GSE87112 was chosen with a default threshold of FDR < 1E-6. A default promoter region window was defined as 250 bp upstream and 500 bp downstream of TSS^37,39^. Using these three gene mapping strategies, we mapped the lo-siRF-prioritized SNV set to 21 protein-coding genes (Fig. 4a), of which 20 are HGNC-recognizable. Each of the 21 genes was also functionally linked to a specific lo-siRF-prioritized LV hypertrophy risk locus (Fig. 2f), to which the highest proportion of SNVs mapped to the given gene were assigned. A Circos plot (Fig. 3a) showing comprehensive information of the lo-siRF-prioritized epistatic interactions, FUMA-prioritized eQTL SNV-to-gene connections and 3D chromatin interactions, as well as LD structures and prioritized genes was created by TBtools^79^. We then submitted these 21 genes to the GENE2FUNC process in FUMA and obtained GTEx gene expression data for 19 (out of the 21) genes across multiple tissue types (Fig. 4b). In addition, we used the same approach and mapped the reference SNV set (mentioned in the Methods section Functional interpretation step 1: Extraction of candidate SNVs and LD structures) to a separate gene set that contains 382 HGNC-approved genes. The lo-siRF-prioritized gene set and the reference gene set were used for gene set enrichment analysis that are described in the following sections.

### Gene ontology and pathway enrichment analysis

Genes that co-associate to shared gene ontology (GO) and pathway terms are likely to be functionally related. To assess the differential GO and pathway co-association among the lo-siRF-prioritized genes relative to their counterparts that were deprioritized by lo-siRF, we performed an integrative GO and pathway enrichment analysis followed by an exhaustive permutation of co-association scores between any possible gene-gene combinations found in the aforementioned 382 HGNC-approved genes (see the Methods section Functional interpretation step 4: Functional gene mapping).

In order to improve GO and pathway prioritization, we adopted the concept from Enrichr-KG^48^ and ChEA3^47^ to assess enrichment analysis results across libraries and domains of knowledge as an integrated network of genes and their annotations. We first queried the 382 HGNC-approved genes from the reference gene set against various prior-knowledge gene set libraries in Enrichr^46^ (https://maayanlab.cloud/Enrichr/). We selected five representative libraries from the GO and pathway Enrichr categories as follows: GO biological process^80,81^, GO molecular function^80,81^, MGI Mammalian Phenotypes^82^, Reactome pathways^83^, and KEGG pathways^84^. Other FUMA-extracted genes that were not approved by HGNC using synonyms or aliases were discarded. This enrichment analysis allowed us to search for a union of enriched GO or pathway terms and their correspondingly annotated gene sets, from which we built a co-association network. According to the method by Enrichr-KG^48^, nodes in the co-association network are either the enriched GO and pathway terms or genes.

To measure the degree of co-association to specific GO and pathway terms for two given interactor genes, we computed a co-association score for each of the 72,771 possible gene-gene combinations (from the 382 queried genes). The co-association score was calculated by $R=N_{\left(A\cap B\right)}/N_{\left(A\cup B\right)}$. Here, $N_{\left(A\cap B\right)}$ denotes the number of GO or pathway terms that were significantly enriched for both *gene A* and *gene B* in the proposed gene-gene combination, and $N_{\left(A\cup B\right)}$ is the number of GO or pathway terms that were enriched for either *gene A* or *gene B*. For cases where $N_{\left(A\cup B\right)}=0$, we defined R=0 to indicate that no GO or pathway terms were found to be co-associated with the respective gene-gene combinations. Of the 382 HGNC-approved genes, 20 genes were mapped to lo-siRF-prioritized loci by FUMA functional gene mapping (one of the 21 lo-siRF-prioritized genes is HGNC-unrecognizable and is discarded). We compared the co-association scores R for gene-gene combinations in the lo-siRF-prioritized gene set relative to the full distribution of R provided by an exhaustive permutation of all possible gene-gene combinations in the set of 382 HGNC-approved genes. The ranking of gene-gene combinations was determined by the two-sided empirical *p*-values. Fig. 4c displays significant co-associations (empirical *p* < 0.05) between enriched GO or pathway terms and genes functionally mapped to lo-siRF-prioritized epistatic and hypostatic loci (Fig. 2f). Further details can be found in Extended Data 6.

### Transcription factor enrichment analysis

Owing to the limitations and biases of various specific assays, we performed an integrative transcription factor (TF) enrichment analysis against multiple annotated gene set libraries in ChEA3^47^ and Enrichr^46^. To preserve the variety of library types, we assembled 9 gene set libraries (Fig. 4d) from distinct sources as follows:

1. Putative TF target gene sets determined by ChIP-seq experiments from ENCODE^85^;
2. Putative TF target gene sets determined by ChIP-seq experiments from ReMap^86^;
3. Putative TF target gene sets determined by ChIP-seq experiments from individual publications^47^;
4. TF coexpression with other genes based on RNA-seq data from GTEx^41^;
5. TF coexpression with other genes based on RNA-seq data from ARCHS4^87^;
6. Single TF perturbations followed by gene differential expression^46^;
7. Putative target gene sets determined by scanning PWMs from JASPAR^88^ and TRANSFAC^89^ at promoter regions of all human genes;
8. Gene sets predicted by transcriptional regulatory relationships unraveled by sentence-based text-mining (TRRUST)^90^;
9. Top co-occurring genes with TFs in a large number of Enrichr queries^47^.

Of the mentioned 9 gene set libraries, libraries 1, 3, and 5 were assembled by combining gene set libraries downloaded from both ChEA3^47^ and Enrichr^46^. Libraries 2, 4, and 9 were downloaded from ChEA3^47^. Libraries 6, 7, and 8 were downloaded from Enrichr^46^. According to the integration method by ChEA3^47^, for libraries in which multiple gene sets were annotated to the same TF, the unique gene set with the lowest FET *p*-value was used. As mentioned in previous sections, we used separate FUMA SNP2GENE processes and functionally mapped lo-siRF-prioritized SNVs and all GWAS-filtered SNVs to a lo-siRF-prioritized gene set (20 HGNC-recognizable genes) and a reference gene set (382 HGNC-recognizable genes), respectively. Because the lo-siRF-prioritized gene set is a subset of the reference gene set, we considered the 362 genes complementary to the lo-siRF-prioritized gene set as the lo-siRF-deprioritized gene set. Taking the 20 lo-siRF-prioritized genes and 263 lo-siRF-deprioritized genes as two separate input gene sets, we performed enrichment analysis against the 9 gene set libraries. For each of the 9 libraries, we ranked the significance of overlap between the input gene set and the TF-annotated gene sets in that library by FET *p*-values. Those TFs with identical FET *p*-values were ranked by the same integer number. A scaled rank was then assigned to each TF by dividing the corresponding integer rank by the maximum integer rank in its respective library. We then integrated the 9 sets of TF rankings and re-ordered the TFs by two sequential criteria: (1) the number of libraries that display a significant overlap with the input gene set (FET *p* < 0.05) and (2) the mean scaled rank across all libraries containing that TF. Using this method, we prioritized two distinct sets of TFs for the lo-siRF-prioritized genes (Fig. 4d, top) and lo-siRF-deprioritized genes (Fig. 4d, bottom).

We used the same approach described in the Methods section Gene ontology and pathway enrichment analysis to evaluate the differential TF co-association between the lo-siRF-prioritized genes relative to the lo-siRF-deprioritized genes. A TF co-association score was computed for each of the 72,771 possible gene-gene combinations from the 382 genes. Still, the TF co-association score was computed by $R=N_{\left(A\cap B\right)}/N_{\left(A\cup B\right)}$, except that $N_{\left(A\cap B\right)}$ and $N_{\left(A\cup B\right)}$ denote the number of enriched TF terms instead of GO or pathway terms. Pairwise interactions between lo-siRF-prioritized genes were extracted and ranked by the empirical *p*-values (Fig. 4e) from an exhaustive permutation of TF co-association scores for the 72,771 possible gene-gene combinations. Details regarding TF enrichment from both lo-siRF-prioritized and lo-siRF-deprioritized genes and TF co-association strengths can be found in Extended Data 7.

### Disease-state-specific gene co-expression network analysis

In order to evaluate the connectivity between genes and their potential roles in the transition from healthy to failing myocardium, we compared gene-gene connectivity and changes in the topological structure between gene co-expression networks for healthy and failing human heart tissues (Fig. 5). To construct gene co-expression networks, cardiac tissue samples from 177 failing hearts and 136 donor, non-failing (control) hearts were collected from operating rooms and remote locations for RNA expression measurements. We performed weighted gene co-expression network analysis (WGCNA) on the covariate-corrected RNA microarray data for the control and heart failure networks separately (Fig. 5a). Detailed steps for generating these co-expression networks, which included calculating the correlation matrix, TOM transformation, and Dynamic Tree Cut module finding, are described in our previous study^51^, and data for these networks is available at https://doi.org/10.5281/zenodo.2600420. To evaluate the degree of connectivity between interacting genes in each of the networks, we compared the edge weights between the interacting genes demonstrated in this study relative to the distribution of all possible pairwise combinations of genes (Fig. 5b and 5c). We also evaluated the difference of edge weights (Z-score normalized) between the control and heart failure networks to understand how these gene-gene interactions change between non-failing and failing hearts (Fig. 5d). The two-tailed empirical *p*-value represents the proportion of the absolute difference in edge weights of all gene pairs that exceed the absolute difference score for gene pairs of interest. We then compared the structure of modules derived from dendrograms on the WGCNA control and heart failure networks (Fig. 5e and 5f). Modules were labeled according to Reactome Enrichment analysis of genes within each module. The full gene module descriptions and Benjamini-Hochberg-adjusted enrichment *p*-values can be found in the Supplementary Data 5 and 6 in the study by Cordero et al.^51^.

### Induced pluripotent stem cell cardiomyocytes differentiation

The studied patient-specific human induced pluripotent stem cells (hiPSCs) were derived from a 45-year-old female proband with a heterozygous *MYH7*-R403Q mutation. Derivation and maintenance of hiPSC lines were performed following Dainis et al.^30^. Briefly, hiPSCs were maintained in MTeSR (StemCell Technologies) and split at a low density (1:12) onto fresh 1:200 matrigel-coated 12 well plates. Following the split, cells were left in MTeSR media supplemented with 1 μM Thiazovivin. The hiPSCs were maintained in MTeSR until cells reached 90% confluency, which began Day 0 of the cardiomyocyte differentiation protocol. Cardiomyocytes were differentiated from hiPSCs using small molecule inhibitors. For Days 0–5, cells were given RPMI 1640 medium + L-glutamine and B27 − insulin. On Days 0 and 1, the media was supplemented with 6 μM of the GSK3β inhibitor, CHIR99021. On Days 2 and 3, the media was supplemented with 5 μM of the Wnt inhibitor, IWR-1. Media was switched to RPMI 1640 medium + L-glutamine and B27 + insulin on Days 6–8. On Days 9–12, cells were maintained in RPMI 1640 medium + L-glutamine − glucose, B27 + insulin, and sodium lactate. On Day 13, cells were detached using Accutase for 7–10 minutes at 37 °C and resuspended in neutralizing RPMI 1640 medium + L-glutamine and B27 + insulin. This mixture was centrifuged for 5 minutes at 1000 rpm (103 rcf). The cell pellet was resuspended in 1 μM thiazovivin supplemented RPMI 1640 medium + L-glutamine and B27 + insulin. For the rest of the protocol (Days 14–40), cells were exposed to RPMI 1640 medium + L-glutamine − glucose, B27 + insulin, and sodium lactate. Media changes occurred every other day on Days 14–19 and every three days for Days 20–40. On Day 40, cardiomyocytes reached maturity.

### RNA silencing in induced pluripotent stem cell-derived cardiomyocytes

Mature hiPSC-derived cardiomyocytes were transfected with Silencer Select siRNAs (Thermofisher) using TransIT-TKO Transfection reagent (Mirus Bio). Cells were incubated for 48 hours with 75 nM siRNA treatments. Four wells of cells were transfected with each of the six siRNAs: scramble, *CCDC141* (ID s49797), *IGF1R* (ID s223918), *TTN* (ID s14484), *CCDC141* and *IGF1R*, and *CCDC141* and *TTN*. After 2 days, hiPSC-CMs were collected for RNA extraction.

### RT-qPCR analysis for siRNA gene silencing efficiency

Following cell morphology measurement, all cells for each condition were centrifuged for 5 minutes at 1000 rpm (103 rcf). Cell pellets were frozen at −80 °C prior to RNA extraction. RNA was extracted using Trizol reagent for RT-qPCR to confirm gene knockdown occurred. Reverse Transcription of RNA was done using High-Capacity cDNA Reverse Transcription Kit (Thermofisher). qPCR of the single stranded cDNA was performed using TaqMan Fast Advanced MM (Thermofisher) with the following annealing temperatures: 95°C 20” and 40 cycles of 95°C 1” and 60°C 20”. qPCR of the silenced genes was performed using TaqMan^®^ Gene Expression Assays, including *CCDC141* (Hs00892642_m1), *IGF1R* (Hs00609566_m1), and *TTN* (Hs00399225_m1). For gene silencing efficiency analysis, gene RPLP0 (Hs00420895_gH) was used as a reference gene. Data were analyzed using the delta-delta Ct method.

### Cell sample preparation for cell morphology measurement

Following siRNA treatments, cells were detached for microfluidic single cell imaging using a mixture of 5 parts Accutase and 1 part TrypLE, treated for 6 minutes at 37 °C. Cells were then added to the neutralizing RPMI 1640 medium + L-glutamine and B27 + insulin. These mixtures were centrifuged for 5 minutes at 1000 rpm (103 rcf). For each gene silencing condition, the four wells of cells were resuspended in 4 mL of the MEM medium, which is composed of MEM (HBSS balanced) medium, 10% FBS, and 1% Pen Strep (Gibco). Cells were filtered with 100 μm strainers (Corning) before adding into the microfluidic devices.

### Microfluidic inertial focusing device

We developed a new spiral inertial microfluidics system on the basis of the study by Guan et al.^28^ to focus randomly suspended cells into separate single streams based on cell size for high-resolution and high-throughput single cell imaging. The microfluidic device (Extended Data Fig. 6) contains 5 loops of spiral microchannel with a radius increasing from 3.3 mm to 7.05 mm. The microchannel has a cross-section with a slanted ceiling, resulting in 80 μm and 150 μm depths at the inner and outer side of the channel, respectively. The channel width is fixed to 600 μm. The 495 μm wide slanted region of the channel ceiling is composed of ten 7 μm deep stairs. This particular geometry induces strong Dean vortices in the outer half of the channel cross-section, leading to high sensitivity of size separation and cell focusing. The device has two inlets at the spiral center to introduce cell suspensions and sheath flow of fresh medium. At the outlet region, the channel is expanded in width and split into two outlet channels with a width of 845 μm for the top outlet and 690 μm for the bottom outlet. Depths of the two outlet channels are designed to create equal hydraulic resistance. The top and bottom outlet channels are connected to 80 μm and 50 μm deep straight observation channels for high-throughput cell imaging.

### Microdevice fabrication

The spiral microchannel was fabricated by CNC micromachining a piece of laser-cut poly (methyl methacrylate) (PMMA) sheet, which was bonded with a PMMA chip machined only with the inlet channels and another blank PMMA chip using a solvent-assisted thermal binding process to form the enclosed channel^26^. Before bonding, PMMA chips were cleaned with acetone, methanol, isopropanol and deionized water in sequence. Droplets of a solvent mixture (47.5% DMSO, 47.5% water, 5% methanol) were evenly spread over the cleaned chips. The PMMA chips were assembled appropriately and clamped using a customized aluminum fixture, and then heated in a ThermoScientific Lindberg Blue M oven at 96 °C for 2 hrs. After bonding, fluid reservoirs (McMaster) were then attached to the chips using a two-part epoxy (McMaster). Microchannels were flushed with 70% ethanol followed by DI water for sterilization.

### High-throughput single cell imaging

Before each experiment, microchannels were flushed with 3 mL of the MEM medium. Prepared cell samples and fresh MEM medium were loaded into 3 mL syringes, which were connected to the corresponding microchannel inlets using Tygon PVC tubing (McMaster). Both cells and the fresh MEM medium were infused into the microchannel using a Pico Plus Elite syringe pump (Harvard Apparatus) at 1.2 mL/min. Microscope image sequences of cells focused to the top and bottom observation channels were captured using a VEO 710S high-speed camera (Phantom) with a sampling rate of 700 fps and a 5 μsec light exposure.

### Image analysis for cell feature extraction

For each gene silencing condition of each biological repeat, 21,000 images were processed to extract cell morphology features. To analyze cell size and shape changes induced by gene silencing, we developed a MATLAB-based image analysis pipeline, which includes three major steps: image preparation, feature extraction, and image post-processing (Fig. 6c). In step one, image sequences were fed into the MATLAB program and subtracted from the corresponding background image to correct any inhomogeneous illumination. The program automatically generates background images, in which each pixel value is computed as the mode pixel intensity value among the same pixel of the entire corresponding image sequence. After illumination correction, step two detects cell edges by looking for the local maxima of the bright field intensity gradient, following which the program closes edge gaps, removes cells connected to the image borders, cleans small features (noise), and then fills holes to generate binary images and centroid positions for each single cell. Cell locations were then traced and stuck cells were removed by a double-counting filter if present. The double-counting filter excludes measurements collected around the same location with similar cell sizes using a Gaussian kernel density method (bandwidth = 0.09) when the estimated density for a certain location and size exceeds a particular threshold. The maximal density value for experimental runs where no repeated measurements were observed was used as the threshold. This procedure was manually validated using visual inspection of the removed cells. Binary images passing the double-counting filter were used to create coordinates (X, Y) of cell outlines, which leads to a range of cell size and shape parameters, including cell diameter and area, solidity, roundness error, circularity, and intensity spatial relationship enclosed within the cell boundary. Cell area was computed as the 2D integration of the cell outline, and the cell diameter was computed as $2\sqrt{\text{Area}/\pi }$. Solidity is the ratio of cell area to the area of the smallest convex polygon that contains the cell region. Roundness error was computed as the ratio between the standard deviation and mean of radii on the cell outline measured from the centroid. Circularity was calculated as $4Area\pi \;/\;Perimeter{\;}^{2}$. The 2D intensity distributions within cell outlines were used to derive peak locations and count peak numbers, which is a measure of intensity spatial relationship and a gating parameter to remove clumped cells. In the post-processing step, data were cleaned using three filters with the following gating threshold. To remove large clumps, the peak-solidity filter removes data outside of the polygonal region defined by {(0.9, 0), (0.9, 3.2), (0.934, 8.26), (1, 28), (1, 0)} in the (solidity, peak No.) space. Then, the roundness filter removes cells with weird shapes by excluding data with a roundness error higher than 0.3 or a circularity lower than 0.6. Finally, the small size filter removes cell debris whose major diameter is lower than 15 μm (12 μm) or minor diameter is lower than 12 μm (10 μm) for images photographed at the top (bottom) outlet microchannels.

### Statistical analysis of experimental results

To analyze the experimental results, we compared differences in cell sizes and morphological features between cells with silenced genes or gene pairs and their scramble controls using various summary statistics. To study the centers of the distributions, we compared the medians using a traditional Wilcoxon signed rank test (Fig. 6f) and a bootstrap quantile test at the 0.5 quantile level. In accordance with the PCS framework, we used two different tests here to ensure that our findings are robust to this arbitrary modeling choice and that the underlying assumptions do not drive our results. We further performed a bootstrap-t for trimmed means of cell size distributions and evaluated the stability of results by varying the amount of trimming (ranging from 0–0.3) to verify that outliers (e.g., from large clumps of cells) do not drive the conclusions (data not shown). In addition to comparisons of central behavior, we performed the bootstrap quantile test across increasing quantile levels (ranging from 0.5–0.9) to study cell size changes (Extended Data Fig. 7) that favor hypertrophic cells, which is more relevant to the pathologic phenotype of cardiac hypertrophy. All tests are performed on each experimental batch, and the maximum *p*-value across batches are reported in the main text. To formally quantify whether interactions are non-additive, for each interaction (i.e.,*CCDC141*-*TTN* and *CCDC141*-*IGF1R*), we tested whether the interaction effect is significant when regressing cell size on the interaction and main effects for each relevant gene. Again in accordance with the PCS framework, we performed a stability analysis by comparing results across different regression parameters – namely, quantile regression with increasing quantile levels (ranging from 0.5–0.9). In each regression, percentile bootstrap t-tests were used to compute *p*-values. Traditional t-test *p*-values supporting these results are also provided in Extended Data 8 as a stability check. Since these regressions require comparisons between gene silencing experiments (e.g., silencing *CCDC141* and *TTN* vs only silencing *CCDC141*), and gene silencing efficiency varied across experiments, batches were merged based on those with the highest efficiency for each regression.

## Extended Data

## Supplementary Material

Supplement 1

## Data Availability

All genotype and cardiac MRI data used as input to the lo-siRF pipeline are available from the UK Biobank (https://www.ukbiobank.ac.uk/). This work was conducted under the UK Biobank application 22282. GWAS-filtered SNVs using PLINK^34^ and BOLT-LMM^35^ are summarized in Extended Data 2. Data for the gene co-expression networks from 313 explanted human hearts is available at https://doi.org/10.5281/zenodo.2600420.
