# Supplementary material for: Epistasis regulates genetic control of cardiac hypertrophy
Source: Res Sq. 2023 Nov 20:rs.3.rs-3509208. Preprint. [Version 1] doi: 10.21203/rs.3.rs-3509208/v1 (PMC10690313; doi:10.21203/rs.3.rs-3509208/v1)
Supplement: Supplement 1 [file NIHPPrs3509208v1-supplement-1.pdf]

Supplementary Tables

Supplementary Table 1: Characteristics of 29,661 analyzed participants in the UK Biobank.

	Female	Male
N	15189	14472
LVMi (g/m^2)	40.7 (5.7)	51.3 (7.8)
LVM (g)	70.7 (12.3)	102.7 (18.6)
Age (y)	63.4 (7.5)	64.9 (7.7)
Height (cm)	162.9 (6.2)	176.2 (6.6)
Weight (kg)	69.0 (13.0)	83.7 (13.3)
Hypertensive Diseases	20.1%	29.8%
Aortic Stenosis	0.1%	0.2%
Heart Failure	0.1%	0.6%
Type II Diabetes	1.6%	3.2%
Blood Pressure Medication	12.1%	20.0%

Summary statistics of the 29,661 unrelated White British individuals analyzed in this study. Means and standard deviations (in parentheses) are reported for continuous measurements (LVMi, LVM, age, height, and weight) alongside the number of individuals (N) and the percentage of individuals with various cardiac hypertrophy-related diseases or on blood pressure medication. We define hypertensive diseases as anyone with self-reported hypertension, high blood pressure as diagnosed by a doctor, or any ICD10 billing code diagnosis in I10-I16; aortic stenosis as self-reported aortic stenosis or an ICD10 billing code diagnosis of I35; heart failure as self-reported heart failure or an ICD10 billing code diagnosis of I50; and type II diabetes as self-reported type II diabetes or an ICD10 billing code diagnosis of E11.

Supplementary Table 2: Thresholds defining low and high LVMi groups used in siRF fit

	Male		Female	
Binarization Threshold	Low LVMi Threshold	High LVMi Threshold	Low LVMi Threshold	High LVMi Threshold
0.15	43.8	58.5	35.1	46.1
0.20	45.1	56.8	36.0	44.9
0.25	46.0	55.4	36.8	43.8

For each of the three binarization thresholds used in lo-siRF (corresponding to the bottom/top 15th, 20th, and 25th quantiles), we provide the gender-specific LVMi cutoffs for the low and high LVMi groups. All thresholds were measured in g/m^2.

Supplementary Table 3: Prediction accuracies of methods across different LVMi binarization thresholds.

	Binarization Threshold = 0.15			Binarization Threshold = 0.20			Binarization Threshold = 0.25		
Method	Accuracy	AUROC	AUPRC	Accuracy	AUROC	AUPRC	Accuracy	AUROC	AUPRC
siRF	0.554	**0.585**	**0.579**	**0.557**	**0.583**	**0.562**	**0.563**	**0.582**	**0.556**
RF	0.546	0.572	0.571	0.553	0.569	0.549	0.554	0.569	0.555
Lasso	0.547	0.559	0.541	0.545	0.556	0.529	0.534	0.550	0.526
Ridge	**0.559**	0.567	0.550	0.539	0.563	0.539	0.541	0.555	0.536
SVM	0.553	0.566	0.552	0.551	0.565	0.541	0.544	0.558	0.541

Maximum prediction accuracies highlighted in bold. The siRF model performs better or on par with other commonly used machine learning methods when predicting the binarized LVMi phenotype. This result holds across all three binarization thresholds and three different classification metrics, i.e., classification accuracy, area under the receiver operator curve (AUROC), and area under the precision-recall curve (AUPRC). In accordance with the prediction check component of the PCS framework, siRF is an appropriate fit for the given data.

Supplementary Table 4: Top signed loci and interactions between loci, prioritized by lo-siRF across LVMi binarization thresholds.

Loci / Interaction	Binarization Threshold 0.15	0.20	0.25	Mean p-value
CCDC141⁻–IGF1R⁻	$< 10^{-3}$	$< 10^{-4}$	$< 10^{-4}$	**$< 10^{-3}$**
IGF1R⁻	$< 10^{-3}$	$< 10^{-3}$	$< 10^{-3}$	**$< 10^{-3}$**
MIR588;RSPO3⁺	0.002	0.004	$< 10^{-4}$	**0.002**
TTN⁻	0.022	0.006	$< 10^{-3}$	**0.009**
CCDC141⁻–TTN⁻	0.030	0.002	$< 10^{-3}$	**0.011**
TTN⁺	0.030	0.005	$< 10^{-3}$	**0.012**
MIR588;RSPO3⁻	0.016	0.014	0.009	**0.013**
LSP1⁻	0.029	0.019	0.002	**0.017**
CCDC141⁻	0.033	0.007	0.015	**0.018**
CCDC141⁻–LOC157273;TNKS⁻	0.099	0.024	0.044	**0.056**

A list of the top signed loci and interactions between loci, prioritized by lo-siRF, that were stably important across all three LVMi binarization thresholds (Supplementary Table 2). These loci and interactions between loci are ranked by the lo-siRF *p*-value, averaged across the three binarization thresholds.

Supplementary Table 5: Summary of siRF evaluation metrics for top interactions between loci.

Threshold	Prevalence	Precision	Class Difference in Prevalence	Stability of Class Difference in Prevalence	Independence of Feature Selection	Stability of Independence of Feature Selection	Increase in Precision	Stability of Increase in Precision	Stability
CCDC141⁻–IGF1R⁻									
Binarization Threshold = 0.15	0.064	0.56	0.012	1.0	0.00094	0.78	0.017	1.0	0.68
Binarization Threshold = 0.2	0.062	0.54	0.0095	1.0	0.0044	1.0	0.014	1.0	0.64
Binarization Threshold = 0.25	0.077	0.54	0.012	1.0	0.011	1.0	0.019	1.0	0.82
CCDC141⁻–LOC157273;TNKS⁻									
Binarization Threshold = 0.15	0.20	0.56	0.040	1.0	0.012	1.0	0.020	1.0	1.0
Binarization Threshold = 0.2	0.19	0.56	0.037	1.0	0.020	1.0	0.028	1.0	1.0
Binarization Threshold = 0.25	0.23	0.55	0.043	1.0	0.0068	1.0	0.022	1.0	1.0
CCDC141⁻–TTN⁻									
Binarization Threshold = 0.15	0.12	0.56	0.023	1.0	0.0066	1.0	0.016	1.0	1.0
Binarization Threshold = 0.2	0.14	0.55	0.024	1.0	0.0042	0.96	0.017	1.0	1.0
Binarization Threshold = 0.25	0.085	0.52	0.0076	1.0	-0.0036	0.020	0.0024	0.88	0.92

Though prediction accuracy is weak (indicated by precision scores close to 0.5), the lo-siRF-prioritized interactions are stable across binarization thresholds and across bootstrap replicates (indicated by all types of stability scores being close or equal to 1). Here, prevalence measures the proportion of high LVMi individuals for which the interaction appears. Precision measures the probability of having high LVMi given that the interaction is active. The class difference in prevalence is the prevalence of the interaction in high LVMi individuals minus the prevalence in low LVMi individuals. Independence of feature selection evaluates whether the interaction is collectively or individually associated with the responses. The stability of each of these metrics evaluates how stable the respective scores are across 50 bootstrap replicates. The overall stability score (last column) is the proportion of times that the interaction is identified by siRF across 50 bootstrapped replicates. Higher scores for each listed metric indicate greater importance.
